# Viral protein R of human immunodeficiency virus type-1 induces retrotransposition of long interspersed element-1

**DOI:** 10.1186/1742-4690-10-83

**Published:** 2013-08-05

**Authors:** Kenta Iijima, Noriyuki Okudaira, Masato Tamura, Akihiro Doi, Yoshikazu Saito, Mari Shimura, Motohito Goto, Akihiro Matsunaga, Yuki I Kawamura, Takeshi Otsubo, Taeko Dohi, Shigeki Hoshino, Shigeyuki Kano, Shotaro Hagiwara, Junko Tanuma, Hiroyuki Gatanaga, Masanori Baba, Taku Iguchi, Motoko Yanagita, Shinichi Oka, Tadashi Okamura, Yukihito Ishizaka

**Affiliations:** 1 Department of Intractable Diseases, Research Institute, National Center for Global Health and Medicine, 1-21-1 Toyama, Shinjuku-ku, Tokyo 162-8655, Japan; 2 Graduate School of Comprehensive Human Sciences, University of Tsukuba, 1-1-1 Ten-nodai, Tsukuba 305-8577, Japan; 3 Department of Laboratory Animal Medicine, Research Institute, National Center for Global Health and Medicine, 1-21-1 Toyama, Shinjuku-ku, Tokyo 162-8655, Japan; 4 Department of Gastroenterology, Research Center for Hepatitis and Immunology, Research Institute, National Center for Global Health and Medicine, 1-7-1 Kohnodai, Ichikawa, Chiba 272-8516, Japan; 5 Department of Tropical Medicine and Malaria, Research Institute, National Center for Global Health and Medicine, 1-21-1 Toyama, Shinjuku-ku, Tokyo 162-8655, Japan; 6 Division of Hematology, Department of Internal Medicine, National Center for Global Health and Medicine, 1-21-1 Toyama, Shinjuku-ku, Tokyo 162-8655, Japan; 7 AIDS Clinical Center, National Center for Global Health and Medicine, 1-21-1 Toyama, Shinjuku-ku, Tokyo 162-8655, Japan; 8 Division of Antiviral Chemotherapy, Center for Chronic Viral Diseases, Graduate School of Medical and Dental Sciences, Kagoshima University, Kagoshima 890-8544, Japan; 9 Department of Nephrology, Graduate School of Medicine, Kyoto University, Shogoin-Kawahara-cho 54, Sakyo-ku, Kyoto 606-8507, Japan; 10 Section of Animal Model, Department of Infectious Diseases, Research Institute, National Center for Global Health and Medicine, 1-21-1 Toyama, Shinjuku-ku, Tokyo 162-8655, Japan; 11 Department of Legal Medicine, Hyogo College of Medicine, 1-1 Mukogawa- cho, Nishinomiya, Hyogo 663-8501, Japan; 12 Kyoto University, Graduate School of Medicine, Medical Innovation Center, Shogoin-Kawahara-cho 53, Sakyo-ku, Kyoto 606-8507, Japan

**Keywords:** HIV-1, Vpr, Blood, Retrotransposition, LINE-1, ORF1

## Abstract

**Background:**

Viral protein R (Vpr), a protein of human immunodeficiency virus type-1 (HIV-1) with various biological functions, was shown to be present in the blood of HIV-1-positive patients. However, it remained unclear whether circulating Vpr in patients’ blood is biologically active. Here, we examined the activity of blood Vpr using an assay system by which retrotransposition of long interspersed element-1 (L1-RTP) was detected. We also investigated the *in vivo* effects of recombinant Vpr (rVpr) by administrating it to transgenic mice harboring human L1 as a transgene (hL1-Tg mice). Based on our data, we discuss the involvement of blood Vpr in the clinical symptoms of acquired immunodeficiency syndrome (AIDS).

**Results:**

We first discovered that rVpr was active in induction of L1-RTP. Biochemical analyses revealed that rVpr-induced L1-RTP depended on the aryl hydrocarbon receptor, mitogen-activated protein kinases, and CCAAT/enhancer-binding protein β. By using a sensitive L1-RTP assay system, we showed that 6 of the 15 blood samples from HIV-1 patients examined were positive for induction of L1-RTP. Of note, the L1-RTP-inducing activity was blocked by a monoclonal antibody specific for Vpr. Moreover, L1-RTP was reproducibly induced in various organs, including the kidney, when rVpr was administered to hL1-Tg mice.

**Conclusions:**

Blood Vpr is biologically active, suggesting that its monitoring is worthwhile for clarification of the roles of Vpr in the pathogenesis of AIDS. This is the first report to demonstrate a soluble factor in patients’ blood active for L1-RTP activity, and implies the involvement of L1-RTP in the development of human diseases.

## Background

*Viral protein R* (Vpr), an accessory gene of human immunodeficiency virus type-1 (HIV-1), encodes a virion-associated nuclear protein of ~15 kDa [1]. Vpr has a variety of biological functions, including cell cycle abnormalities at the G_2_/M phase and apoptosis of T cells and neuronal cells (for a recent review, see ref. [2]). Notably, it was shown that Vpr was present in the blood of HIV-1-positive patients [3], and we previously reported that 20 of 52 blood samples from HIV-1-positive patients examined were positive for Vpr [4]. Blood Vpr was detected in patients with high titres of HIV-1 and, interestingly, was also detected in patients with low viral titres [4]. On the other hand, purified recombinant Vpr protein (rVpr) functions as a trans-acting factor [5,6], and rVpr activated viral replication in latently infected cells by increasing production of interleukin-6 (IL-6) by monocytes [7]. Further analyses revealed that rVpr-induced IL-6 production depended on p38, a mitogen-activated protein kinase (MAPK), and CCAAT/enhancer-binding protein β (C/EBP-β) [7]. These observations suggest that blood Vpr could induce various clinical symptoms, but it remained unclear whether blood Vpr is biologically active.

Long interspersed element-1 (LINE-1, L1) and Alu are major endogenous retroelements, accounting for ~17 and ~10% of the human genome, respectively [8,9]. As an autonomous retroelement, L1 can retrotranspose not only itself but also other retroelements, such as Alu and SVA (short interspersed element-variable number tandem repeat-Alu, SINE-VNTR-Alu). Intriguingly, a single human cell contains more than 5 × 10^5^ copies of L1, 80–100 of which are competent for retrotransposition (L1-RTP) [10]. During early embryogenesis, L1-RTP incidentally disrupts gene structures, leading to the development of inborn errors [11,12]. Of note, approximately 100 types of inheritable diseases have been identified as sporadic cases caused by mutagenic RTP of L1 or Alu [12]. Although most studies of L1-RTP have focused on early embryogenesis [13-16], recent lines of evidence suggest that L1-RTP is also induced in somatic cells [17-20]. In tumors of epithelial-cell origins and hepatomas, *de novo* L1 insertions were detected in the vicinity of tumor suppressor genes, suggesting that L1-RTP is actively involved in carcinogenesis [21,22]. Because L1-RTP alters cellular properties by causing various genetic alternations, including gene deletions [23,24], DNA damage [25], apoptosis [26] and immune responses [27], deregulation of L1-RTP in somatic cells likely functions as a trigger of various diseases.

Here we present evidence that Vpr is active for induction of L1-RTP, and further demonstrate that 6 of 15 blood samples from HIV-1 patients were positive for Vpr-induced L1-RTP. Interestingly, rVpr reproducibly induced L1-RTP in various organs, including the kidney, when administered to mice that harbored human L1 as a transgene (hL1-Tg mice) [28,29]. Clinically, HIV-1-associated nephropathy (HIVAN), which is mainly observed among African-Americans [30], is an end-stage renal deficiency that is found without apparent correlation with the viral load [31,32]. In view of reports that Vpr is a candidate molecule responsible for HIVAN [33,34], we propose that monitoring blood levels of Vpr is important for determining its involvement in the pathogenesis of HIVAN.

## Results

### rVpr induces L1-RTP

**Figure 1:**
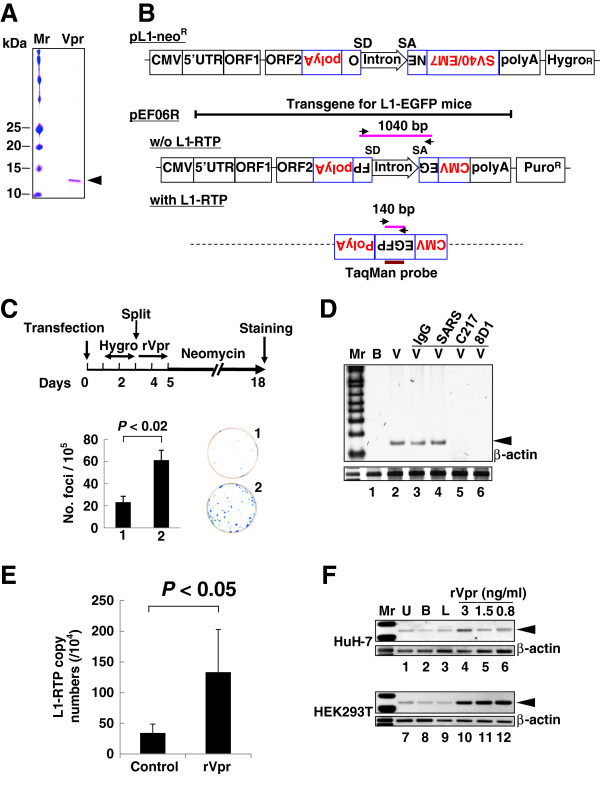
**rVpr induces L1-RTP. A**. rVpr was purified by two-step column chromatography using a glutathione-bead and an affinity column with 8D1. Purified protein was stained with Coomassie brilliant blue. Mr, molecular weight marker. **B**. Schematics of constructs used in the current study (see details in Methods). The PCR-based assay detects a 140 bp band that was amplified upon induction of L1-RTP (with L1-RTP), whereas it detects a 1040 bp band without L1-RTP (w/o L1-RTP). Arrows indicate primers for the PCR-based assay. SD and SA indicate splicing donor and splicing acceptor, respectively. The position of the TaqMan probe was also depicted. **C**. Colony formation assay of rVpr-induced L1-RTP. The experimental protocol and results are shown. HuH-7 cells were treated with buffer (plate no. 1) or rVpr (plate no. 2) are also shown. Obtained colonies were stained (right panels). **D**. Inhibition of rVpr-induced L1-RTP by mAbs against Vpr. 8D1 or C217 were used (lanes 5 and 6). As control, mouse IgG (lane 3) or a SARS mAb (lane 4) were included. B, buffer; V, rVpr. Arrowhead indicates the 140 bp band. Mr, molecular weight marker. **E**. Results of the qPCR analysis of rVpr-induced L1-RTP. Approximately 10 ng/mL of rVpr was used, and L1-RTP was measured by the qPCR. **F**. Activity of low dose of rVpr on HEK293T cells. Results of HuH-7 cells and HEK293T cells were shown. U, untreated; B, buffer; L, LPS (10 ng/mL).

We initially performed a colony formation assay using purified rVpr and pCEP4/L1*mneo*I/ColE1 (pL1-Neo^R^) (Figure 1A and B) [28,35-37]. When HuH-7 human hepatoma cells were treated with rVpr, L1-RTP occurred in approximately 50 of 10^5^ cells (Figure 1C, *P* < 0.02). rVpr caused no apparent cytotoxicity (Additional file 1: Figure S1). The activity of rVpr was also confirmed by a PCR-based assay using pEF06R [37,38], in which the signal intensity of the 140 bp band, which corresponds to a product of L1-RTP, was increased by treatment with rVpr (Figure 1B, lower panel for the rationale of the PCR-based assay and 1D, lane 2). A quantitative PCR (qPCR) analysis was also carried out using a TaqMan probe designed to detect a junction point of two exons of the *EGFP* gene (Figure 1B, bottom; see also Additional file 2: Figure S2 for standard qPCR curves). Data revealed that rVpr significantly increased the frequency of L1-RTP (Figure 1E, *P* < 0.05). Notably, rVpr-induced L1-RTP was completely blocked by 8D1 and C217, monoclonal antibodies (mAbs) against Vpr (Figure 1D, lanes 5 and 6) [4], but not by an irrelevant mAb against a spike protein of severe acute respiratory syndrome coronavirus (Figure 1D, lane 4, SARS). Vpr-induced L1-RTP was also observed in HEK293T cells, in which the activity of ~1 ng/mL rVpr was detected (Figure 1F, lanes 10–12; Additional file 3: Figure S3).

**Figure 2:**
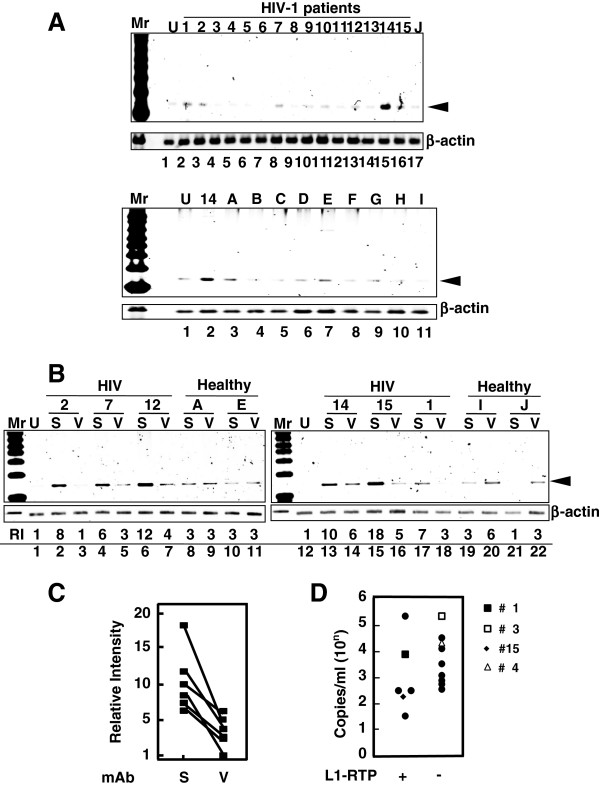
**Detection of Vpr-induced L1-RTP in blood samples of HIV-1-positive patients. A**. Upper panel. Activity for the induction of L1-RTP in the blood of HIV-1-positive patients. Results of the PCR-based assay were shown. Lower panel. As a control, samples of nine healthy volunteers were included (A–I). U, Untreated. **B**. A mAb against Vpr blocked the activity in serum samples. Serum sample of 300 μL was treated with ~500 ng 8D1 (V) or SARS-mAb (S). Serum samples from healthy volunteers were also included (Healthy, A, E, I and J). RI, relative intensity. **C**. Effects of 8D1 on the activity of L1-RTP. RI shown in Figure 2B was plotted and compared. S, treatment with a mAb to SARS; V, 8D1. 8D1 considerably attenuated the L1-RTP-inducing activity in the patients’ blood. **D**. Detection of Vpr-induced L1-RTP in patients with lower viral titres with (+) or without (−) L1-RTP activity. According to the presence of the activity of L1-RTP in blood, patients were separated into two groups. Then, viral loads of each patient were plotted. Blood samples of two patients of each group were subjected to the IP-WB analysis. Vpr was detected in one patient (no. 15, ◆) (Additional file 5: Figure S4). Vpr was not detected in the sample of patient no. 1 (■), who was positive for L1-RTP. Other two patients were negative for both the activity of L1-RTP and Vpr (patient no. 3 and 4, □ and △).

Taking advantage of the high sensitivity of the PCR-based assay performed using HEK293T cells, we explored the activity of L1-RTP in blood samples from HIV-1-positive patients. Among 15 samples analyzed by a PCR assay, 6 were positive for L1-RTP induction (Figure 2A, upper panel; patients’ clinical information is summarized in Additional file 4: Table S1). Notably, L1-RTP activity was selectively blocked by 8D1, indicating that the L1-RTP activity in HIV-1 patients is attributable to Vpr (Figure 2B and C). Interestingly, Vpr-induced L1-RTP was detected in patients with low HIV-1 titres (Figure 2D and Additional file 4: Table S1). To confirm this, we carried out immunoprecipitation followed by Western blot analysis (IP-WB analysis), and successfully detected Vpr in one of two blood samples that were positive for L1-RTP (no. 15; Additional file 5: Figure S4, arrowhead). Estimated concentration of the blood Vpr, when compared to the signals of standard rVpr, would be approximately 5 ng/mL (Additional file 5: Figure S4). In contrast, we could not detect Vpr in another sample (no. 1).

### rVpr induces L1-RTP *in vivo*

**Figure 3:**
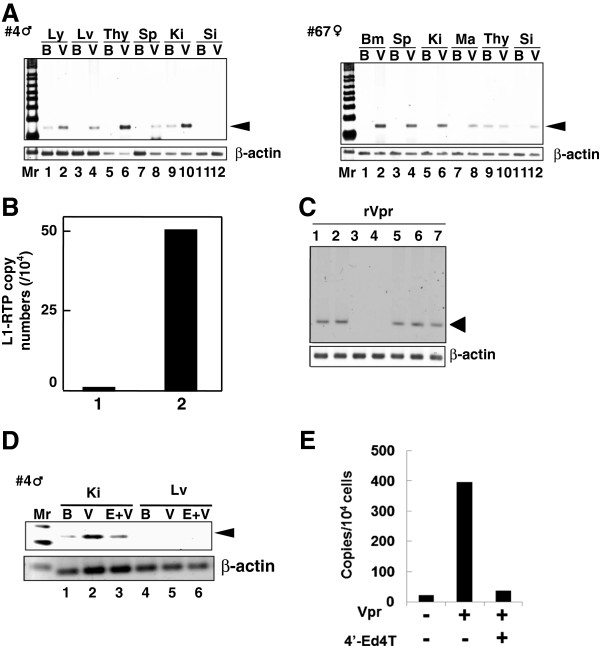
**L1-RTP induction by rVpr *in vivo*. A**. Induction of L1-RTP after intraperitoneal injection of rVpr. rVpr (200 ng; three injections every 2 days) was administered intraperitoneally into two strains of hL1-Tg mice (#4 and 67). On day 2 after the last injection, DNA was extracted from each organ and subjected to the PCR-based assay. Ly, lymph node; Lv, liver; Thy, thymus; Sp, spleen; Ki, kidney; Si, small intestine; Bm, bone marrow; Ma, mammary gland; B, buffer; V, rVpr. Arrowhead indicates L1-RTP. Mr, molecular weight marker. **B**. Effects low dose of rVpr. hL1-Tg mice (#4) were injected six-times with buffer (lane 1) or 10 ng rVpr (lane 2), and DNA extracted from kidney was subjected to the qPCR analysis. **C**. RTIs blocked rVpr-induced L1-RTP. Effects of RTIs (25 μM each) on rVpr-induced L1-RTP was examined. Lane 1, 10 ng/mL rVpr + lamivudine; lane 2, rVpr + AZT; lane 3, rVpr + d4T; lane 4, rVpr + tenofovir; lane 5, rVpr + nevirapine; lane 6, rVpr + efavirenz, lane 7, rVpr + saquinavir. **D**. RTIs inhibited rVpr-induced L1-RTP *in vivo*. Two hours before intravenous administration of 250 ng rVpr, 50 μmoles of 4′-Ed4T was injected intraperitoneally. Lanes 1 and 4, buffer (B); lanes 2 and 5, rVpr (V); lanes 3 and 6, rVpr + 4′-Ed4T (E); Ki, kidney; Lv, liver. **E**. Results of qPCR assay. Similar experiments with Figure 2D were done, and L1-RTP was measured by the qPCR.

To determine the effects of rVpr *in vivo*, we next investigated L1-RTP after administration of rVpr to hL1-Tg mice (Figure 1B, solid line). As shown in Figure 3A, L1-RTP was detected in organs including the lymph nodes, liver, thymus and spleen upon intraperitoneal administration of ~200 ng of rVpr three times every 2 days (Additional file 6: Table S2). Interestingly, the qPCR analysis detected L1-RTP in the kidney after six intravenous administrations of 10 ng of rVpr (Figure 3B). To demonstrate that rVpr-induced L1-RTP was dependent on the reverse transcriptase activity of ORF2 [9], we first carried out *in vitro* experiments to examine whether rVpr-induced L1-RTP was blocked by nucleotide analogue inhibitors of reverse transcriptase (RTIs) [39,40]. As shown in Figure 3C, stavudine (d4T) and tenofovir inhibited the rVpr activity for L1-RTP induction (lanes 3 and 4), but lamivudine (3TC) and azidothymidine (AZT) did not (lanes 1 and 2). The inhibitory effects of d4T on rVpr-induced L1-RTP were potent, and the compound could effectively block the induction of L1-RTP at a concentration of 5 μM (Additional file 7: Figure S5). We next investigated the effects of 2′,3′-didehydro-3′-deoxy-4′-ethynylthymidine (4′-Ed4T), a stavudine analogue with more specific activity as an RTI and fewer side effects [41]. As shown in Figure 3D, 50 μmoles of 4′-Ed4T, when administered intraperitoneally 2 h before intravenous administration of 250 ng of rVpr, efficiently attenuated L1-RTP (compare lanes 2 and 3). qPCR analysis also clearly showed the inhibitory effects of 4′-Ed4T (Figure 3E).

**Figure 4:**
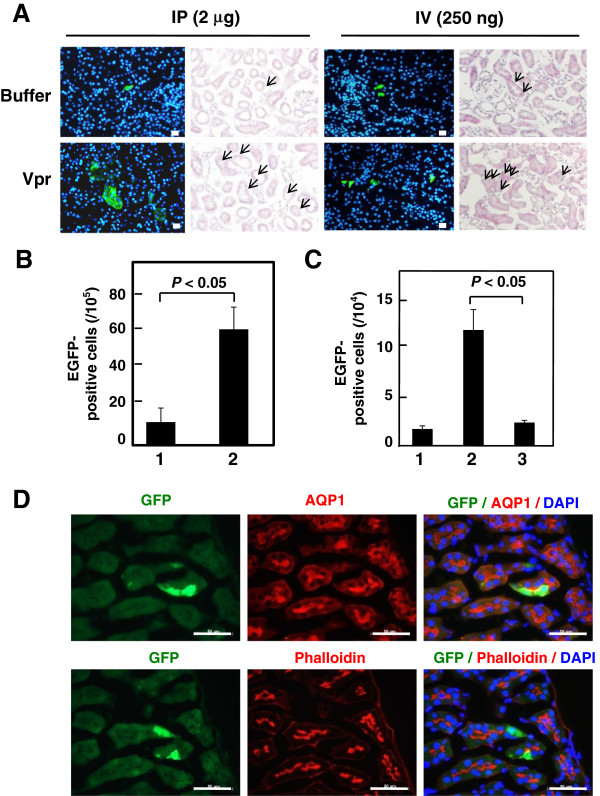
**rVpr induces L1-RTP in proximal RTECs. A**. Detection of rVpr-induced L1-RTP in kidneys. Immunohistochemical analysis using α-GFP was performed. hL1-Tg mice were administered once with 2 μg rVpr intraperitoneally (left four panels) or 250 ng rVpr intravenously (right panels). Upper panels, buffer control; lower panels, rVpr. Green, EGFP-positive cells; blue, Hoechst 33258 staining. Histology after H/E staining was also depicted. Bar, 20 μm (×200). Arrow, EGFP-positive cells. **B.** Induction of L1-RTP in kidney. Results after six times intravenous injections of 10 ng rVpr were shown. Column 1, buffer control; column 2, rVpr. For each sample, three different slices were prepared and the immunohistochemical analysis was done. Obtained numbers of EGFP-positive cells were then subjected to statistical analysis. *P* < 0.05. **C**. rVpr induced L1-RTP was blocked by 4′-Ed4T. Effects of 4′-Ed4T on the induction of L1-RTP by rVpr were examined using #4 hL1-Tg mice. Mice were intravenously injected with 250 ng rVpr six times. To examine the effects of 4′-Ed4T, the compound of 50 μmoles was intraperitoneally injected 2 h prior to injection of rVpr. The inhibitory effects of 4′-Ed4T were statistically significant (*P* < 0.05). Column 1, buffer; column 2, 250 ng rVpr; column 3, 250 ng rVpr + 4′-Ed4T. **D**. rVpr induced L1-RTP in proximal RTECs. Double staining with α-AQP1 or α-phalloidin was performed. Bar, 50 μm (×400). hL1-Tg mice were intravenously injected with 10 ng rVpr six times. In this experiment, no EGFP-positive cells were detected in the control kidney of mouse that was injected with buffer.

By immunohistochemical analysis using α-GFP, we successfully detected cells positive for the induction of L1-RTP after a single injection of 2 μg or 250 ng of Vpr (Figure 4A). Intriguingly, L1-RTP occurred at a frequency of several cells per 10^4^ cells after six administrations of 10 ng of rVpr (Figure 4B, *P* < 0.05). Co-administration of 4′-Ed4T significantly blocked L1-RTP induced by repetitive injection of 250 ng of rVpr (Figure 4C, column 3). Consistent with the results obtained by hematoxylin-eosin (H/E) and α-GFP staining, dual staining for α-aquaporin-1 or α-phalloidin, which are markers of proximal renal tubular cells [42-44], detected rVpr-induced L1-RTP in renal tubular epithelial cells (RTECs) (Figure 4D).

We also investigated the methylation status of CpG in the L1-5′UTR in the rVpr-treated kidney. Analysis by the COBRA method [45], a method of quantifying CpG methylation, detected no apparent changes in the methylation status of CpG before or after six administrations of 10 ng of rVpr (Additional file 8: Figure S6).

### rVpr-induced L1-RTP depends on an AhR-p38-C/EBP-β cellular cascade

**Figure 5:**
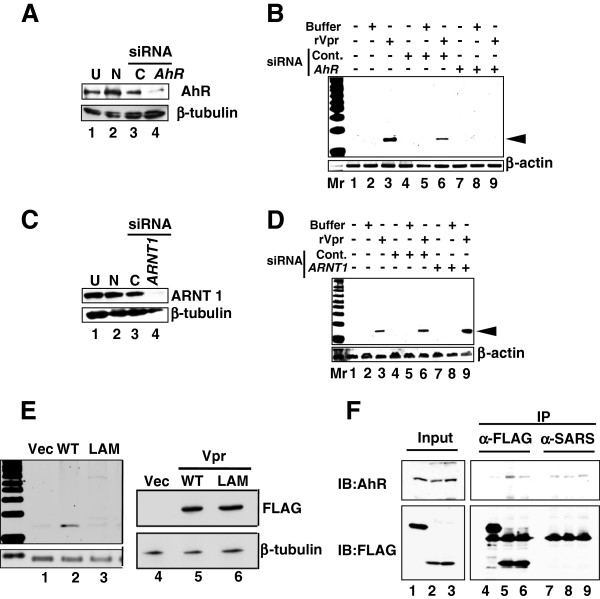
**AhR is required for rVpr-induced L1-RTP. A**. *AhR* siRNA down-regulated expression of endogenous protein. Lane 1, untreated (U); lane 2, mock transfection without siRNA (N); lane 3, control siRNA (C); lane 4, *AhR* siRNA. **B**. *AhR* siRNA attenuated rVpr-induced L1-RTP. **C**. *ARNT1* siRNA down-regulated expression of endogenous ARNT1 protein. Lane 1, untreated (U); lane 2, mock transfection without siRNA (N); lane 3, control siRNA (C); lane 4, *ARNT1* siRNA. **D**. ARNT1 is dispensable for L1-RTP induction by rVpr. **E**. No induction of L1-RTP by the LA mutant. Left panel. Result of L1-RTP after transfection of plasmid DNA encoding wild-type Vpr (WT) or the LA mutant. Lanes 1 and 4, vector control (Vec); lanes 2 and 5, WT Vpr; lanes 3 and 6, LA mutant (LAM). Right panel. Expression levels of WT Vpr and the LA mutant were comparable. **F**. Association with AhR was impaired in the LA mutant. HEK293T cells were transfected with constructs expressing FLAG-EGFP, FLAG-Vpr-Wt or FLAG-Vpr-LAM. Cell extracts were subjected to IP with α-FLAG followed by WB using α-AhR (upper panel) or α-FLAG (lower panel). Lanes 1, 4 and 7, vector control (FLAG-EGFP); lanes 2, 5 and 8, FLAG-Vpr-Wt; lanes 3, 6 and 9, FLAG-Vpr-LAM. α-SARS mAb was used for control-IP.

Previously, we reported that various environmental compounds induced L1-RTP in a manner dependent on the aryl hydrocarbon receptor (AhR), which has been shown to associate with other cellular molecules via an LxxLL motif in the counterpart molecule (amino acids denoted by single letters) [46]. Interestingly, Vpr contains an LQQLL motif at amino acids 64–68 that functions as a sequence motif for binding to host cellular proteins, including p300/histone acetyl transferase [47]. Based on these observations, we hypothesized that AhR functions as a cellular factor responsible for rVpr-induced L1-RTP. To prove this, we first assessed the effects of 3′-methoxy-4′-nitroflavone (MNF), an AhR inhibitor [48], and observed that 10 μM MNF completely blocked rVpr-induced L1-RTP (Additional file 9: Figure S7). Moreover, down-regulation of endogenous AhR expression by *AhR* siRNA was accompanied by reduced rVpr-induced L1-RTP (see Figure 5A, lane 4, and 5B for a representative result from experiments using two different *AhR* siRNAs; see also Additional file 10: Figure S8A and B for data obtained using another *AhR* siRNA). By contrast, down-regulation of ARNT1 by siRNA (Figure 5C) did not attenuate L1-RTP (Figure 5D, lane 9 and Additional file 10: Figure S8D, lane 9). AhR and ARNT1 form a heterodimer (AHR complex) and are involved in the induction of *CYP1A1* mRNA expression in response to environmental pollutants [49]. Both *AhR* and *ARNT1* siRNAs blocked the induction of *CYP1A1* mRNA expression by 6-formylindolo[3,2-*b*]carbazole (FICZ), a tryptophan photoproduct (Additional file 11: Figure S9), indicating that each siRNA efficiently inhibited the functional properties of the AHR complex, further suggesting that rVpr-induced L1-RTP depends on AhR, but not ARNT1.

To determine the importance of the LxxLL motif of Vpr for the induction of L1-RTP, we investigated the activity of a Vpr mutant containing AQQAA instead of LQQLL (LA mutant, “LAM” in Figure 5). First, studies of forced expression of wild-type Vpr (WT Vpr) and the LA mutant revealed that the mutant was not active for induction of L1-RTP (Figure 5E, left panel), although comparable levels of each protein were detected (Figure 5E, right panel). Additionally, IP-WB analysis detected an association between WT-Vpr and AhR (Figure 5F, lane 5), but less interaction of the LA mutant with AhR (Figure 5F, lane 6). These data suggest that Vpr-induced L1-RTP is dependent on a molecular interaction with AhR via the LxxLL motif of Vpr.

**Figure 6:**
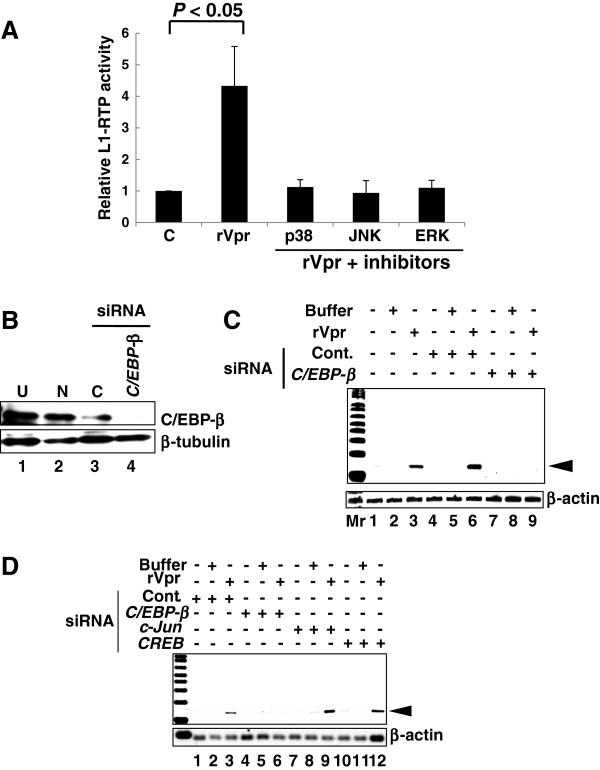
**Involvement of MAPK in rVpr-induced L1-RTP. A**. Inhibition of rVpr-induced L1-RTP by MAPK inhibitors. Before addition of rVpr, SB202190, SP600125 and PD98059, which were inhibitors of p38, JNK and ERK, respectively, were added. Results of the qPCR assay was shown. **B**. Expression of endogenous C/EBP-β is reduced by siRNA application. See also Additional file 10: Figure S8E showing results obtained by different siRNA. Lane 1, untreated (U); lane 2, mock transfectant (N); lane 3, control siRNA (C); lane 4, *C/EBP-β* siRNA. **C**. Inhibition of rVpr-induced L1-RTP by *C/EBP-β* siRNA. Mr, molecular weight marker. **D**. c-Jun and CREB were dispensable for rVpr induced L1-RTP. rVpr induced L1-RTP was investigated after the introduction of siRNAs targeting c-Jun and CREB. C/EBP-β siRNA was included as positive control. This experiment was done using *C/EBP-β* siRNA different from that used in Figures 6B and C. Effects of each siRNA on the expression of endogenous proteins were depicted in Additional file 10: Figures S8F and S8G.

To identify additional cellular factors involved in rVpr-induced L1-RTP, we investigated the involvement of MAPK, because our previous work revealed that Vpr induced IL-6 production via activation of p38 [7]. First, qPCR analysis revealed that the MAPK inhibitors attenuated rVpr-induced L1-RTP to the basal level observed after treatment with control buffer (Figure 6A, see also Additional file 12: Figure S10 for representative qPCR data). Data indicate that the tested compounds inhibited the up-regulation of L1-RTP by rVpr.

In a previous study, we showed that p38 and C/EBP-β are important for understanding the cellular response to exogenously applied rVpr [7], implying that these molecules are also involved in the induction of L1-RTP by rVpr. To confirm this, we focused on the effect of *C/EBP-β* siRNA on rVpr activity. As shown in Figure 6B, transfection of the *C/EBP-β* siRNA down-regulated the endogenous protein level and attenuated rVpr-induced L1-RTP (Figure 6C, lane 9; see also Additional file 10: Figure S8E for data obtained using another siRNA targeting *C/EBP-β* mRNA, which was used in the experiment shown in Figure 6D). In contrast, siRNAs against *CREB* and *c-Jun* did not attenuate rVpr-induced L1-RTP (Figure 6D), although each siRNA efficiently down-regulated endogenous protein expression (Additional file 10: Figure S8F and G). One possible reason is that MAPK inhibitors are not specific for target molecules [37].

### Chromatin recruitment of ORF1 induced by rVpr is dependent on AhR

**Figure 7:**
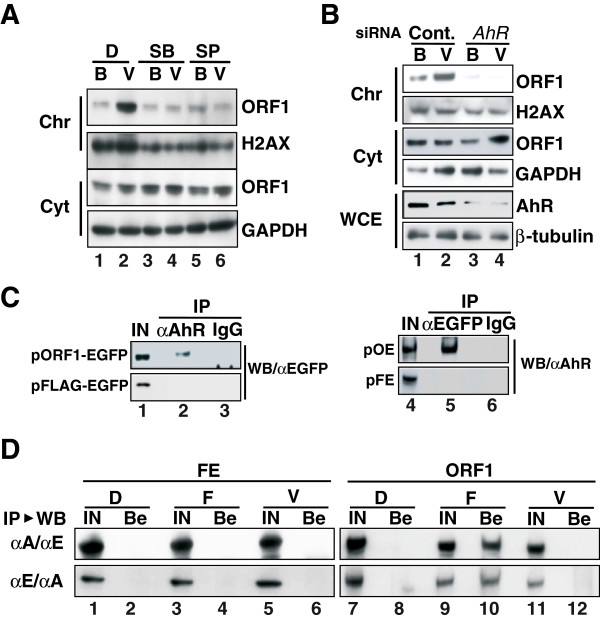
**rVpr-induced chromatin recruitment of ORF1. A**. Chromatin recruitment of ORF1 is dependent on MAPK. HuH-7 cells were transfected with pORF1-TAP, and then WB was performed using a peroxidase-conjugated human IgG, α-H2AX and α-GAPDH. Cellular fractions of chromatin (Chr) and the cytoplasm (Cyt) were analyzed. D, DMSO; SB, SB202190; SP, SP600125; B, buffer; V, rVpr (5 ng/mL). **B**. *AhR* siRNA blocked rVpr-induced ORF1 chromatin recruitment. After transfection of *AhR* siRNA, similar experiments to those shown in Figure 7A were conducted. Chr, chromatin fraction; Cyt, cytoplasmic fraction; WCE, whole-cell extracts; B, buffer; V, rVpr. See also Additional file 13: Figure S11 showing results of independent experiments done using a different *AhR* siRNA. **C**. Association of AhR and ORF1. HuH-7 cells were transfected with constructs expressing a chimeric protein of ORF1 and EGFP (pORF1-EGFP) or a FLAG-tagged EGFP (pFLAG-EGFP). IP-WB was then performed using α-AhR followed by WB using α-EGFP (lane 2). As a reverse experiment, IP using α-EGFP was performed followed by WB using α-AhR (lane 5). **D**. rVpr induced no association of ORF1 and ARNT1. HuH-7 cells were transfected with pFLAG-EGFP (FE) (lanes 1–6) or pORF1-EGFP (ORF1) (lanes 7–12). After treatment of cells with rVpr (lanes 5, 6, 11, and 12) or FICZ (lanes 3, 4, 9 and 10), IP followed by WB were performed using α-ARNT1 and α-EGFP, respectively (upper panels). As a reverse experiment, IP using α-EGFP followed by WB using α-ARNT1 were performed (lower panels). D, DMSO; F, FICZ (10 nM); V, rVpr (5 ng/mL); IN, input; Be, fraction recovered using protein-G beads.

L1 encodes two proteins, open reading frame-1 (ORF1) and ORF2, which are ~40 and ~150 kDa, respectively, and are present in cytoplasmic ribonucleoprotein complexes and cytoplasmic stress granules [50,51]. Moreover, L1-RTP is initiated by target-primed reverse transcription within the genome [9], and ORF1 functions as a nucleic acid chaperone during L1-RTP [52]. These observations suggest that ORF1 is recruited to the chromatin fraction in response to rVpr treatment. To demonstrate chromatin recruitment of ORF1, we transfected a plasmid DNA that encodes ORF1 into HuH-7 cells, and then carried out WB analysis of the chromatin fraction of the transfected cells after treatment of rVpr. The rVpr-induced chromatin recruitment of ORF1 was blocked by MAPK inhibitors examined (Figure 7A, lanes 4 and 6) and the *AhR* siRNA (Figure 7B, lane 4; see also Additional file 13: Figure S11 for results from an independent experiment performed using a different *AhR* siRNA). To further show that ORF1 and AhR form a complex, we transfected a plasmid DNA encoding a chimeric protein of ORF1 and EGFP (pORF1-EGFP) into HuH-7 cells, and then performed IP-WB analysis. IP using α-AhR followed by WB analysis using α-EGFP revealed that ORF1 and AhR were associated (Figure 7C, lane 2). The reverse experiment, in which IP using α-EGFP was followed by WB using α-AhR, confirmed formation of this complex (Figure 7C, lane 5). The interaction between ORF1 and AhR was also detected in cells in which both ORF1 and ORF2 proteins were expressed exogenously (Additional file 14: Figure S12).

Previously, we reported that FICZ is a potent activator of L1-RTP, and that its activity was dependent on ARNT1, but not on AhR [37]. To determine the functional link between ORF1 and ARNT1, we performed IP-WB analysis after transfecting pORF1-EGFP into HuH-7 cells. ORF1 was detected in an extract of cells treated with FICZ and was recovered using α-ARNT1 (Figure 7D, upper panel, lane 10). By contrast, it was not recovered from extracts of cells treated with rVpr using α-ARNT1 (Figure 7D, upper panel, lane 12). Consistent results were obtained in a reverse experiment, in which WB using α-ARNT1 was performed on a sample recovered by IP using α-EGFP (Figure 7D, lower panel). In this case, the cell extract obtained after FICZ treatment yielded a positive signal (Figure 7D, lower panel, lane 10). These data suggest that the association between ORF1 and ARNT1 is induced by exogenous FICZ but not rVpr.

## Discussion

### rVpr-induced L1-RTP depends on an AhR-p38-C/EBP-β cellular cascade

Here we found that Vpr is a viral protein active for the induction of L1-RTP. Experiments using MNF, siRNAs targeting *AhR* and *C/EBP-β* mRNAs, and MAPK inhibitors revealed that rVpr-induced L1-RTP depends on an AhR-p38-C/EBP-β cellular cascade (Figures 5 and 6). We confirmed by *in vitro* experiments that rVpr did not increase expression of *L1* mRNA or the splicing efficiency of an immature *EGFP* transcript derived from the reporter L1 construct (Additional file 15: Figure S13). Moreover, no apparent changes in the CpG methylation status were observed in the 5′UTR of the exogenous hL1 gene in the kidneys of hL1-Tg mice that had been treated with rVpr (Additional file 8: Figure S6). Our data suggest that rVpr-induced L1-RTP is controlled at the post-transcriptional level, although it has been proposed that L1-RTP is influenced at the transcriptional level by the methylation status of the L1-5′UTR [53,54].

In addition to the LA mutant, we investigated the activity of a Vpr mutant lacking the C-terminal 12 amino acids (ΔC12). A PCR-based assay revealed that the ΔC12 mutant was not active for the induction of L1-RTP (Additional file 16: Figure S14). It has been shown that Vpr has an affinity for nucleic acids, which is attributable to the basic moiety in the C-terminal region of Vpr [55]. To exclude the possibility that the induction of AhR-dependent L1-RTP by Vpr depends on binding to nucleic acids, we investigated the interaction between Vpr and AhR after nuclease treatment. IP-WB analysis combined with treatment with benzonase, a nuclease that degrades both DNA and RNA, revealed that their interaction was not reduced (Additional file 17: Figure S15A). Additionally, ORF1 and AhR constitutively formed a complex, and their interaction was also resistant to nuclease treatment (Additional file 17: Figure S15B). Moreover, rVpr triggered chromatin recruitment of ORF1 in an AhR-dependent manner (Figure 7B). Taken together, these data suggest that Vpr functions as an AhR ligand, and activates a cellular cascade for the induction of L1-RTP.

### Biologically active Vpr is present in the blood of HIV-1-positive patients

We detected L1-RTP-inducing activity in the blood of HIV-1 patients: 6 of 15 patients were positive for the induction of L1-RTP (Figure 2A). The L1-RTP activity in those six patients was selectively blocked by 8D1, a mAb against Vpr (Figure 2B). Interestingly, we previously examined blood Vpr by IP-WB analysis, and detected Vpr in 20 of 52 blood samples from HIV-1 patients [4]. Interestingly, the positive frequencies observed in these two sets of experiments are comparable, but greater numbers of samples are needed to conclude that blood Vpr is exclusively biologically active. Although it was reported that an antibody against Vpr is present in patients’ blood [56], and implied that Vpr activity would be blocked by such autoantibodies, our current experiments proved that blood Vpr is active for the induction of L1-RTP. Because L1-RTP can alter cellular properties by inducing DNA damage and apoptosis [9], it is tempting to speculate that blood Vpr can modify clinical outcomes of AIDS patients.

Consistent with our previous observation that Vpr protein was detectable in blood samples from HIV-1-positive patients with low viral titres [4], we here detected Vpr-induced L1-RTP in samples from patients with low viral titres. As shown in Figure 2, L1-RTP-inducing activity was detected in some blood samples, and IP-WB analysis successfully detected a Vpr signal (no. 15) (Additional file 4: Table S1 and Additional file 5: Figure S4). Intriguingly, however, the viral titre of patient 15 was 140 copies/mL (Figure 2D, closed diamond and Additional file 4: Table S1). By striking contrast, the viral titres of the blood samples from patients 3, 8 and 4 were >10^4^ copies/mL, but no apparent L1-RTP-inducing activity was detected. Although it remains completely unknown why Vpr was present in patients with low viral titres, one possible explanation would be that Vpr is secreted into the blood from latent foci in patients. *In vitro* experiments support the notion that Vpr is excreted by infected cells and functions as a soluble protein with bystander effects [57].

### rVpr is active for the induction for L1-RTP *in vivo*

Repeated intravenous administration of 10 ng of rVpr, a dose comparable to patients’ blood levels [7], induced L1-RTP *in vivo* (Figure 4B). We observed that administration of rVpr induced L1-RTP in various organs, such as the lymph nodes and spleen. Additionally, we found that Vpr also induced L1-RTP in the kidney (Figure 4A and Additional file 6: Table S2). Notably, even a single injection of 250 ng of rVpr into the tail vein induced L1-RTP in the kidneys (Figure 4A, right panels), suggesting that the kidney is a target organ of Vpr-induced L1-RTP. Immunohistochemical analysis showed that Vpr induced L1-RTP in RTECs, especially in proximal RTECs (Figure 4D). Previously, it was reported that Vpr and Nef are candidate mediators of HIVAN: forced expression of these viral genes in mouse podocytes resulted in proteinuria and glomerular diseases [34]. Although it was proposed that renal dysfunction is a direct effect of primary HIV-1 infection in RTECs [58], it remains to be investigated whether repeated administration of rVpr causes renal insufficiency.

HIVAN develops mostly in people of African descent, and shows the strong influence of genetic traits [59-61]. However, its mechanism remained completely unknown. Importantly, HIVAN has no apparent correlation to viral load [31], and, intriguingly, it has been proposed that the kidneys are a latent reservoir of HIV-1 [62,63]. Based on these observations, it is plausible that both blood-circulating Vpr and Vpr secreted locally from a latent reservoir (the kidney, for example) attack RTECs. To prove this, further studies are required to determine whether the kidney is an organ from which Vpr is constitutively secreted.

In addition to their clinical relevance for HIV-1 pathogenesis, our findings should have a general impact on the involvement of L1-RTP in human diseases. By analysis of tumors using second-generation sequencing technology, *de novo* L1 insertions were detected in the vicinity of tumor suppressor genes [21,22], suggesting that L1 insertion was actively involved in carcinogenesis. Additionally, it was shown that de-regulation of L1-RTP is positively linked to the development of autoimmune diseases [27]. Although these lines of evidence revealed that L1-RTP is induced in somatic cells and is involved in the development of human diseases, it remained unclear how L1-RTP is induced in somatic cells. It was previously reported that 2-amino-1-methyl-6-phenylimidazo[4,5-*b*]pyridine (PhIP), a food-borne carcinogen, induced L1-RTP in the mouse mammary gland, a target organ of carcinogenesis, when it was administered orally to hL1-Tg mice [29]. Given that PhIP is present in broiled meat [64] and has been detected in human breast milk [65], it is plausible that humans are susceptible to the induction of L1-RTP by environmental factors. Further study is required to demonstrate the activity of L1-RTP under pathological conditions, enabling the roles of L1-RTP in disease development to be specified.

## Conclusions

Six of the 15 blood samples from HIV-1-positive patients examined were positive for Vpr-induced L1-RTP. L1-RTP-inducing activity was detected in blood samples with low viral titres. Monitoring circulating Vpr in relation to clinical outcomes is important to clarify the roles of Vpr in AIDS symptoms. The present study is the first to show that L1-RTP-inducing activity is present *in vivo*, shedding light on the possible involvement of L1-RTP in human diseases. In further research, it will be important to detect L1-RTP-inducing activity under pathological conditions.

## Methods

### Chemicals and cells

HuH-7 cells (RCB1366) and HEK293T cells (RCB2202) were obtained from the Riken BioResource Centre Cell Bank. They were cultured in Dulbecco’s modified Eagle’s medium supplemented with 10% fetal calf serum (Sigma-Aldrich, St. Louis, MI, USA). The transfection efficiencies were ~70 and ~30%, respectively, as determined by fluorescence-activated cell sorting (FACS) on day 2 after transfection of plasmid DNA encoding enhanced green fluorescent protein (EGFP) (data not shown). MNF was kindly provided by Dr. Gabriele Vielhaber (Symrise, Holzminden, Germany). SB20358, SP60012, PD98059 and lipopolysaccharide (L8274) were from Sigma-Aldrich. FICZ was obtained from Enzo Life Sciences (Plymouth Meeting, PA, USA). Protease inhibitors (Roche Diagnostics, Tokyo, Japan) were also purchased.

Antibodies against AhR, (Santa Cruz Biotechnology, Santa Cruz, CA, USA), ARNT1 (Santa Cruz Biotechnology), β-tubulin (Thermo Fisher Scientific, Waltham, MA, USA), H2AX (Millipore, Billerica, MA, USA), C/EBP-β (Cell Signaling Technology Inc., Danvers, MA, USA), FLAG (Sigma-Aldrich), EGFP (rabbit antibody: Medical & Biological Laboratories, Co., Ltd., Nagoya, Japan; mouse monoclonal antibody: Abcam, Cambridge, United Kingdom), aquaporin 1 (AQP1; Abcam) and glyceraldehyde 3-phosphate dehydrogenase (GAPDH; Trevigen, Gaithersburg, MD, USA) were used as the primary antibodies. A rabbit polyclonal antibody against human ORF1 was generated using the peptide MGKKQNRKTGNSKTQSAC (amino acids denoted by single letters) as an immunogen (Medical & Biological Laboratories). An Alexa Fluor 546-conjugated antibody to phalloidin (Invitrogen, Carlsbad, CA, USA) was purchased. As secondary antibodies, α-mouse IgG (GE Healthcare Bio-Sciences Corp., Piscataway, NJ, USA), α-rabbit IgG (GE Healthcare), and α-goat IgG (Santa Cruz Biotechnology), all of which were conjugated with horseradish peroxidase, were used. For immunohistochemical analysis, Alexa Fluor 555-conjugated goat α-rabbit IgG (Invitrogen) and Alexa Fluor 488 goat α-mouse IgG (Invitrogen) were used as the secondary antibodies. Hoechst 33258 was purchased from Invitrogen.

Based on recent reports that RTIs efficiently blocked L1-RTP [39,40], we used 4′-Ed4T, which has more potent inhibitory activity than d4T and less effect on DNA polymerases, and which is currently undergoing phase IIb clinical trials in HIV-1-infected patients [41]. Two hours before injection of rVpr, 50 μmoles of 4′-Ed4T was injected intraperitoneally to give a final concentration of approximately 25 μM when most of the compound is transferred to the blood, the estimated volume of which is ~2 mL.

### Purification of rVpr and assays of L1-RTP

rVpr was expressed using pGEX-6P-1 in *Escherichia coli* BL21 and purified as described previously (Figure 1A) [4]. Purified rVpr was tested for endotoxin using a highly sensitive lipopolysaccharide (LPS) assay with Limulus amebocyte lysate, the detection limit of which was 0.25 EU/mL (Wako Pure Chemical Ind., Ltd., Osaka, Japan). For L1-RTP assays, we used two reporter constructs, pEF06R [38] and pCEP4/L1mneoI/ColE1 (pL1-Neo^R^) [28,35-37], for semi-quantitative and quantitative PCR, and colony formation assays, respectively. Each construct contained all components of human L1 with single transcriptional units with EGFP or Neo^R^, which were inserted in reverse orientation. When L1-RTP occurs, the intron within each reporter gene is spliced out, and then pEF06R expresses functional EGFP, whereas pL1-Neo^R^ expresses a functional neomycin-resistance gene (Neo^R^). Cells were transfected with pEF06R or pL1-Neo^R^ using Lipofectamine 2000 (Invitrogen) or Xfect (Takara Bio Inc., Shiga, Japan). Cells were selected for 2 days with puromycin (Puro, 0.5 μg/mL) for pEF06R, or with hygromycin (Hygro, 25 μg/mL) for pL1-Neo^R^. Next, cells were treated for additional 2 or 3 days with the indicated amounts of rVpr.

For the PCR assay, genomic DNA was prepared from harvested cells using a DNA extraction system (QuickGene; Fujifilm, Tokyo, Japan). For semi-quantitative PCR, primers that were designed for each exon would amplify a product of ~1040 bp, whereas they would generate a product of ~140 bp upon L1-RTP. Thus, occurrence of L1-RTP was determined by evaluating the size of the amplified product [28,29,37]. After staining of amplified DNA with SYBR Green I (LONZA, Basel, Switzerland), signal intensities of the 140 bp bands were measured using a molecular imager (FX-PRO; Bio-Rad, Hercules, CA, USA) and normalized by the signal intensity of the β-actin band, used as the internal control. Relative intensities (RIs) of each 140 bp band were calculated by standardizing the signal of the buffer-treated sample as “1”.

For qPCR analysis, 5′-GAA CGG CAT CAA GGT GAA CT-3′ and 5′-GGG GTG TTC TGC TGG TAG TG-3′, which were designed for each exon of the *EGFP* gene, were used as forward and reverse primers, respectively. A TaqMan-probe (5′-FAM- TGC AG * C TGG CCG AC -MGB-3′) (Invitrogen) was used to detect an amplicon of 87 bp in length (* denotes the exon junction). Template DNA was amplified with Eagle Taq Master Mix (Roche Diagnostics) and a CFX Connect Real-Time System (Bio-Rad) using the following amplification conditions: 95°C for 10 min, followed by 45 cycles of 95°C for 15 sec and 64°C for 15 sec. To obtain a standard curve for EGFP-qPCR, *EGFP* DNA generated after the induction of L1-RTP was amplified using the above primers and cloned into the pGEM-T Easy vector (Promega, Madison, USA). After confirmation of the nucleotide sequence, standard samples were prepared by mixing human or mouse genomic DNA with the *EGFP*-containing plasmid to give 1.0, 10^-1^, 10^-2^, 10^-3^ and 10^-4^ copies/cell. To normalize the amounts of input DNA, human β-globin or mouse β-actin was quantified by qPCR with SYBR Premix Ex Taq (TaKaRa) and the CFX Connect Real-Time System (Bio-Rad). For human β-globin, the forward primer was 5′-TTG GAC CCA GAG GTT CTT TG-3′ and the reverse primer was 5′-GAG CCA GGC CAT CAC TAA AG-3′; for mouse β-actin, the forward primer was 5′-TGA CGT TGA CAT CCG TAA AGA CC-3′ and the reverse primer was 5′-AAG GGT GTA AAA CGC AGC TCA-3′.

In the colony formation assay, ~2.0 × 10^6^ cells were transfected with pL1-Neo^R^ and selected with 25 μg/mL Hygro, and 1.0 × 10^5^ cells were re-plated to new plates (Split). Next, cells were treated for 2 days with rVpr and further cultured in the presence of neomycin (800 μg/mL) [35-37]. In the initial experiment, we used 5–10 ng/mL rVpr because the maximum reported plasma Vpr concentration in HIV-1-positive patients is ~5 ng/mL [4]. To determine Vpr activity for L1-RTP induction, each of the six plates was treated with rVpr or a buffer control for 2 days, and further cultured in the presence of neomycin. After 3–4 weeks, cell aggregates were stained with methylene blue, and colonies were enumerated. To minimize plate-to-plate variation, the colony numbers of the middle four of the six plates were subjected to statistical analysis. At least two independent experiments were performed, representative results of which are shown.

### Suppression of rVpr-induced L1-RTP by mAbs against Vpr

The effects of mAbs against Vpr on the induction of L1-RTP were investigated by applying 5 ng of rVpr with 500 ng of 8D1 and C217 [4], giving an approximately 10-fold excess molar amount of rVpr. After 60 min of incubation at room temperature, a 300 μL reaction mixture was filtrated and added to 1.5 mL of culture medium of cells. As a control, a SARS mAb, an irrelevant mAb that recognizes a spike protein of the severe acute respiratory syndrome corona virus (SARS-CoV), was used.

### Effect of down-regulation of endogenous proteins on induction of L1-RTP

For each gene, two small interfering RNAs (siRNAs) were prepared (Applied Biosystems, Foster City, CA, USA or Thermo Scientific), and their functions were evaluated by transfection into cells followed by WB analysis. The nucleotide sequences of each siRNA are shown in Additional file 18: Table S3. To evaluate the inhibitory effects of the siRNAs on L1-RTP induction, each siRNA was introduced on day 3 after initial transfection with pL1-Neo^R^ or pEF06R. Two days later, the cells were re-plated, incubated for 2 days with rVpr, and subjected to analysis. Silencer Negative Control siRNAs (cat. no. AM4613, AM4637 and AM464; Life Technologies Corporation, Carlsbad, CA, USA) were used as controls.

### Effects of MAP kinase inhibitors on rVpr-induced L1-RTP

HuH-7 cells were transfected with pEF06R and selected for 2 days with 0.5 μg/mL Puro. On day 3 after transfection, cells were re-plated and subjected to an L1-RTP assay. To examine the effects of MAPK inhibitors, SB202190, SP600125 and PD98059 at concentrations of 1, 100 and 20 μM, respectively, were added 1 h before the addition of rVpr. The cells were exposed to 10 ng/mL rVpr for 3 days and subjected to qPCR analysis. Genomic DNA was isolated using the QuickGene DNA Tissue Kit S and QuickGene-800 (Fujifilm). To selectively detect *EGFP* genes derived from L1-RTP, ~250 ng of DNA were used as the qPCR template. To amplify *β-globin* gene as an internal control, ~50 ng of DNA were used as the qPCR template.

### Administration of rVpr to hL1-Tg mice and L1-RTP assessment

For *in vivo* experiments, we used two transgenic mouse lines, #4 and #67, in which the L1-DNA fragment of pEF06R had been introduced as a transgene (hL1-Tg mice; Figure 1A, sidebar) [28,29]. These two lines were selected because they display low background L1-RTP during embryogenesis but respond vigorously to environmental compounds [29]. The CpG island of the 5′ untranslated region of introduced human L1 (L1-5′UTR) was highly methylated in #4 and #67 mice, as assessed by a PCR-based assay using methylation-specific primers [29]. All animal experiments were approved by the Animal Care and Use Committee at the National Center for Global Health and Medicine (NCGM).

### Clinical samples

Fifteen blood samples obtained from anti-retroviral therapy-naïve male patients who presented to the NCGM hospital between October 1996 and October 2003 were subjected to the PCR-based assay. The patients were 21–44 years of age with viral loads and CD4 counts of 50–230,000 copies/mL and 315–795 cells/mL, respectively. Nine healthy volunteers served as controls. To detect L1-RTP-inducing activity, HEK293T cells were first transfected with pEF06R and selected with 0.5 μg/mL Puro. Then, 150 μL of each heat-inactivated patient serum sample was added to 1.35 mL of culture medium of HEK293T cells. To show that L1-RTP activity in patients’ blood was attributable to Vpr, 100 μL of serum was reacted for 60 min with 500 ng of 8D1 or SARS-S mAb at room temperature in a 300 μL reaction volume. The experimental protocol was approved by the institutional review board of NCGM.

### L1-RTP activity of the Vpr mutant

The LA mutant, which contains AQQAA at codons 64–68, and wild-type (WT) Vpr were expressed as FLAG-tagged proteins using the pFLAG-CMV2 expression vector (Sigma-Aldrich). To obtain comparable levels of expression of each protein, the molar ratio of 1:4 of plasmid DNA for the wild-type Vpr and the LA mutant were transfected respectively. On the next day of transfection, cells were subjected to the PCR-based assay.

### Chromatin recruitment of ORF1 induced by rVpr

We used the pORF1-TAP (tandem affinity purification) construct [66], which encodes a chimeric protein of ORF1, protein A and calmodulin-binding protein. On day 2 after transfection of pORF1-TAP into HuH-7 cells, 5 ng/mL rVpr was added to the culture medium, and cell extracts were prepared on the following day. The chromatin-enriched fraction (chromatin fraction) was isolated using a Subcellular Protein Fractionation Kit (Thermo Fisher Scientific) with micrococcal nuclease, as described previously [29]. Detection of ORF1-TAP was performed by probing with a horseradish peroxidase-conjugated human IgG (Jackson ImmunoResearch West Grove, PA, USA). H2AX was used as an internal control for the chromatin fraction.

### ORF1, AhR, and Vpr complex formation

HuH-7 or HEK293T cells were transfected with the plasmid constructs pFLAG-Vpr-Wt or pFLAG-Vpr-LA mutant, pORF1-EGFP and pFLAG-EGFP, which encode FLAG-tagged Vpr, a chimeric protein of ORF1 and EGFP, and FLAG-tagged EGFP, respectively. On day 2 after transfection, cells were treated with 10 ng/mL rVpr for 1 day to evaluate the dependence of the protein-protein interaction on Vpr. Then, cells were subjected to IP-WB analysis. To analyze the ORF1-AhR association, cells were suspended in a buffer containing 50 mM Tris (pH 7.5), 150 mM NaCl, 1% NP40, 1 mM EDTA and a protease inhibitor cocktail and subjected to brief sonication. For analysis of the Vpr-AhR association, cells were suspended in a buffer containing 25 mM HEPES (pH 7.5), 200 mM NaCl, 0.1% NP40, 10% glycerol and a protease inhibitor cocktail, and were completely lysed by passage through 22 G and 27 G needles (in that order) ten times. Cell extracts (500 to 2000 μg) were pre-cleared with protein G Sepharose beads (GE Healthcare), reacted with 4 μg of α-AhR, α-EGFP, α-FLAG or α-SARS, and then recovered with protein G beads. As an “input” sample, about 5 or 10% of each extract subjected to immunoprecipitation, was assessed simultaneously.

### Immunohistochemical analysis of EGFP-positive cells

After perfusion fixation, organs were immersed in 0.1 M phosphate buffer (PB) (pH 7.4) supplemented with 4% paraformaldehyde at 4°C. On the following day, samples were serially immersed at 4°C in PB supplemented with 10% saccharose for 1 h, 20% saccharose until immersed completely, and then 30% saccharose overnight. Next, samples were embedded in Optimal Cutting Temperature compound (Sakura Finetek, Torrance, CA, USA) for cryosectioning. Using a cryostat (Leica Biosystems, Wetzlar, Germany), three slices (5 μm thick) were prepared from different sections of the fixed kidney: a first slice from the middle part of the kidney, a second section is from the part that contained mainly cortex with little amount of medulla, and the third section that is composed mainly of cortex. Samples were washed three times with 0.1 M phosphate-buffered saline (PBS) (10 min per wash), and incubated for 30 min at room temperature in Image-iT Fx signal enhancer (Invitrogen). After rinsing three times with 25 mM Tris-HCl (pH 7.5), 150 mM NaCl, and 0.05% Tween 20 (TBST) (10 min each), sections were then reacted with rabbit α-EGFP antibody (1:2000; Medical & Biological Laboratories) in TBST supplemented with 1% bovine serum albumin (BSA) at 4°C. On the following day, specimens were rinsed three times with TBST, and then incubated with Alexa Fluor 555-conjugated goat α-rabbit IgG antibody (1:5000; Invitrogen) for 2 h at room temperature. Nuclear DNA was stained with Hoechst 33258 at a final concentration of 0.36 μM. Fluorolabeled sections were examined under a fluorescence microscope (Olympus BX51; Olympus, Tokyo, Japan). Using the cellSens system (Olympus), total cell numbers in each section were first counted automatically. Next, numbers of EGFP-positive cells were counted manually. The frequency of EGFP-positive cells was calculated using the numbers of total and EGFP-positive cells. Three independent sections were prepared from a single specimen and subjected to analysis. The significance of the frequency of EGFP-positive cells was then evaluated statistically.

To identify RTECs positive for L1-RTP, immunohistochemistry was performed as described previously [42] using the following primary antibodies; α-GFP antibody (1:200 dilution) (Abcam, UK), α-AQP1 antibody (1:200 dilution) [43], and Alexa Fluor 546-phalloidin (1:400 dilution) (Invitrogen) [44].

### L1-5′UTR methylation status

We performed sodium bisulfite treatment of genomic DNA using the EZ DNA Methylation Kit (Zymo Research, Irvine, CA, USA), according to the manufacturer’s instructions. One microlitre of the aliquot was used as the template for combined bisulfite restriction analysis (COBRA) [45]. Primers used for amplification of the L1 transgene promoter region were as follows: forward 5′-GTAAGGGGTTAGGGAGTTTTT-3′ and reverse 5′-CCTTACAATTTAATCTCAAACTA-3′. The PCR reactions were performed in a volume of 20 μL containing 1 μL of bisulfite-treated genomic DNA, primers (0.3 μM each), and a 10 μL EpiTect MSP Kit (Qiagen, Hilden, Germany). The amplification conditions consisted of 40 cycles of 94°C for 15 sec, 50°C for 30 sec and 72°C for 30 sec. PCR products were digested using the restriction enzyme *Taq* I (New England Biolabs, Ipswich, MA, USA), which is specific for the methylated sequence, after sodium bisulfite treatment. Digested products were resolved by 3% agarose gel electrophoresis and stained with ethidium bromide.

### Statistical analysis

Statistical significance was evaluated using the Mann-Whitney U-test. A *P* value < 0.05 was deemed to indicate statistical significance.

## Competing interest

All authors declare that they have no competing interest for the current work.

## Authors’ contributions

NO, MT, YS, KI, MS, AD and SH carried out biochemical analyzes using cell lines. KY, TO and TD NO, MT, YS, MG, AD and TO performed experiments using hL1-Tg mice. YK, TO, KI and YS established qPCR of L1-RTP. AM and NO analyzed methylation status of CpG in the L1-5′UTR. YS, NO, MT, TI and MY carried out immunohistochemistry of cells positive for rVpr-induced L1-RTP. NO, SH, JT, HG and SO analyzed correlation of the activity of Vpr-induced L1-RTP in blood of HIV-positive patients and clinical manifestations. NO, MB and MT examined the effects of RTIs on rVpr-induced L1-RTP. SK and YI designed experiments. NO, KI, MT, AD, YS and YI were involved in preparation of the manuscript. All authors read and approved the final manuscript.

## Supplementary Material

Additional file 1: Figure S1 No cytotoxicity of rVpr. Click here for file

Additional file 2: Figure S2 Standard curve of qPCR assay with a TaqMan probe. Click here for file

Additional file 3: Figure S3 L1-RTP induced by low dose of rVpr. Click here for file

Additional file 4: Table S1 Summary viral titres and L1-RTP activity. Click here for file

Additional file 5: Figure S4 Detection of Vpr in blood samples of HIV-1 positive patients. Click here for file

Additional file 6: Table S2 Summary of the PCR-based assay *in vivo*. Click here for file

Additional file 7: Figure S5 Effects of d4T on rVpr-induced L1-RTP. Click here for file

Additional file 8: Figure S6 No changes of methylation status of CpG in the L1-5′UTR. Click here for file

Additional file 9: Figure S7 Inhibitory effects of MNF on rVpr-induced L1-RTP. Click here for file

Additional file 10: Figure S8 Effects of siRNAs of *AhR, ARNT1*, *CREB* and c-*Jun* on expression of endogenous proteins. Click here for file

Additional file 11: Figure S9 *CYP1A1* expression under down-regulation of AhR or ARNT1. Click here for file

Additional file 12: Figure S10 Effects of MAPK inhibitors on rVpr-induced L1-RTP. Click here for file

Additional file 13: Figure S11 Effects of AhR siRNA on chromatin recruitment of ORF1. Click here for file

Additional file 14: Figure S12 Constitutive association of ORF1 and AhR under the conditions competent for the induction of L1-RTP. Click here for file

Additional file 15: Figure S13 No apparent changes of expression of L1 mRNA after the addition of rVpr. Click here for file

Additional file 16: Figure S14 L1-RTP by Vpr required a carboxy-terminal region. Click here for file

Additional file 17: Figure S15 Effects of benzonase on the interaction of AhR and ORF1 or Vpr. Click here for file

Additional file 18: Table S3 Nucleotide sequence of siRNA used in the current study. Click here for file
