# Supplementary material for: Viral protein R of human immunodeficiency virus type-1 induces retrotransposition of long interspersed element-1
Source: Retrovirology. 2013 Aug 5;10:83. doi: 10.1186/1742-4690-10-83 (PMC3751050; doi:10.1186/1742-4690-10-83)
Supplement: Additional file 1: Figure S1 — No cytotoxicity of rVpr. [file 1742-4690-10-83-S1.ppt]

## Slide 1
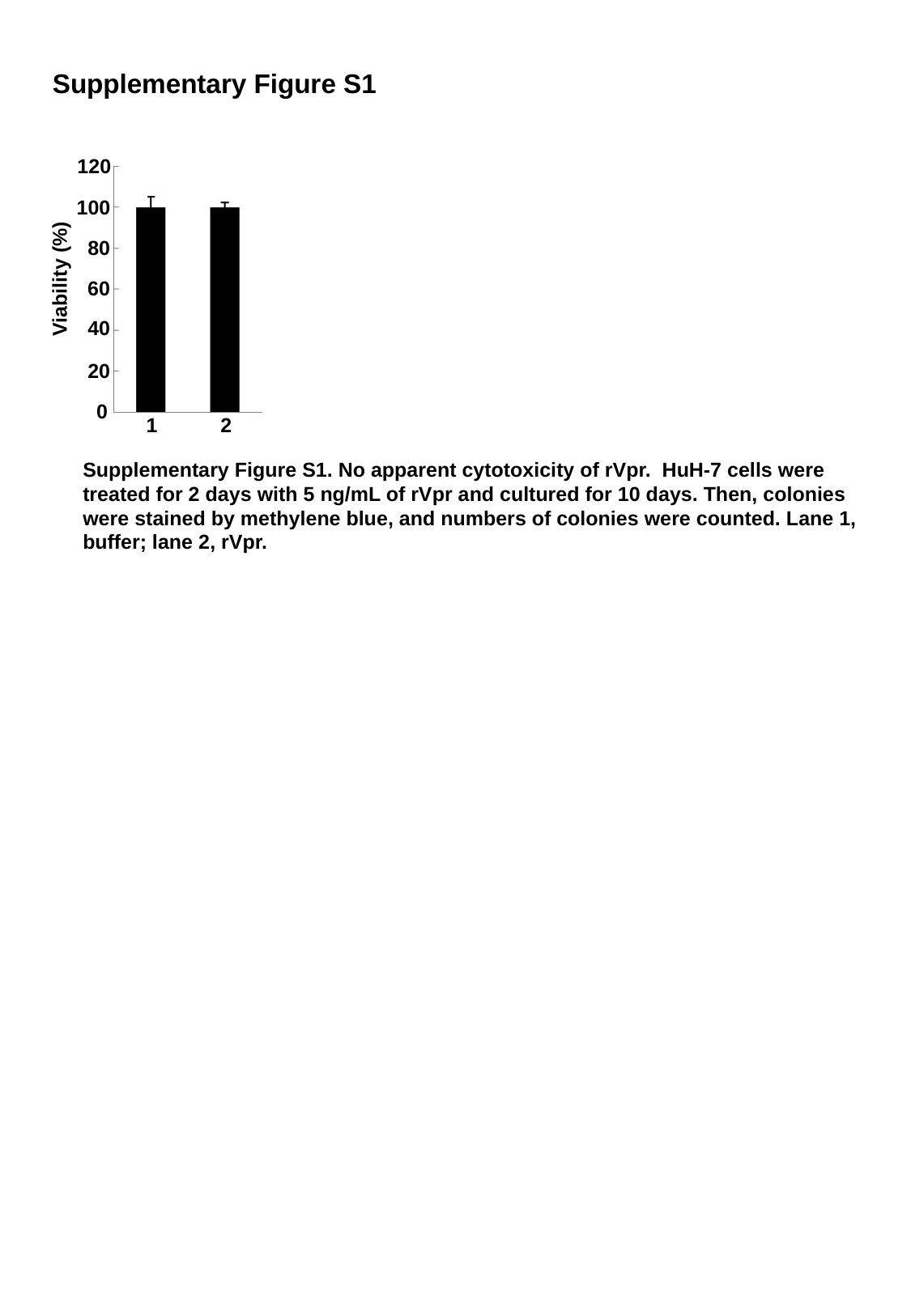

Supplementary Figure S1
120
100
80
Viability (%)
60
40
20
0
1
2
Supplementary Figure S1. No apparent cytotoxicity of rVpr. HuH-7 cells were treated for 2 days with 5 ng/mL of rVpr and cultured for 10 days. Then, colonies were stained by methylene blue, and numbers of colonies were counted. Lane 1, buffer; lane 2, rVpr.
