# Supplementary material for: Viral protein R of human immunodeficiency virus type-1 induces retrotransposition of long interspersed element-1
Source: Retrovirology. 2013 Aug 5;10:83. doi: 10.1186/1742-4690-10-83 (PMC3751050; doi:10.1186/1742-4690-10-83)
Supplement: Additional file 2: Figure S2 — Standard curve of qPCR assay with a TaqMan probe. [file 1742-4690-10-83-S2.ppt]

## Slide 1
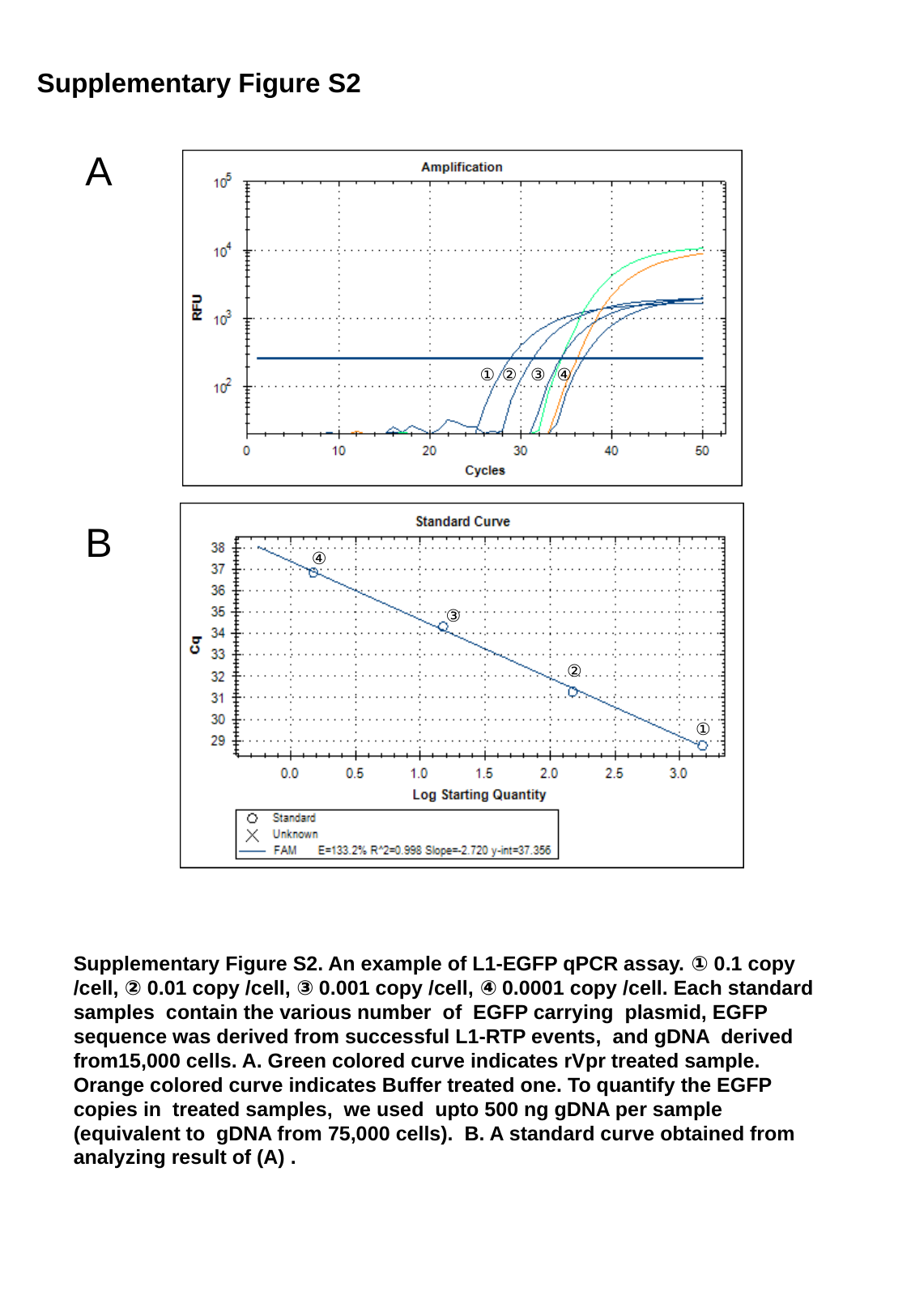

Supplementary Figure S2
A
①
②
③
④
④
③
②
①
B
Supplementary Figure S2. An example of L1-EGFP qPCR assay. ① 0.1 copy /cell, ② 0.01 copy /cell, ③ 0.001 copy /cell, ④ 0.0001 copy /cell. Each standard samples contain the various number of EGFP carrying plasmid, EGFP sequence was derived from successful L1-RTP events, and gDNA derived from15,000 cells. A. Green colored curve indicates rVpr treated sample. Orange colored curve indicates Buffer treated one. To quantify the EGFP copies in treated samples, we used upto 500 ng gDNA per sample (equivalent to gDNA from 75,000 cells). B. A standard curve obtained from analyzing result of (A) .
