# Supplementary material for: Viral protein R of human immunodeficiency virus type-1 induces retrotransposition of long interspersed element-1
Source: Retrovirology. 2013 Aug 5;10:83. doi: 10.1186/1742-4690-10-83 (PMC3751050; doi:10.1186/1742-4690-10-83)
Supplement: Additional file 3: Figure S3 — L1-RTP induced by low dose of rVpr. [file 1742-4690-10-83-S3.ppt]

## Slide 1
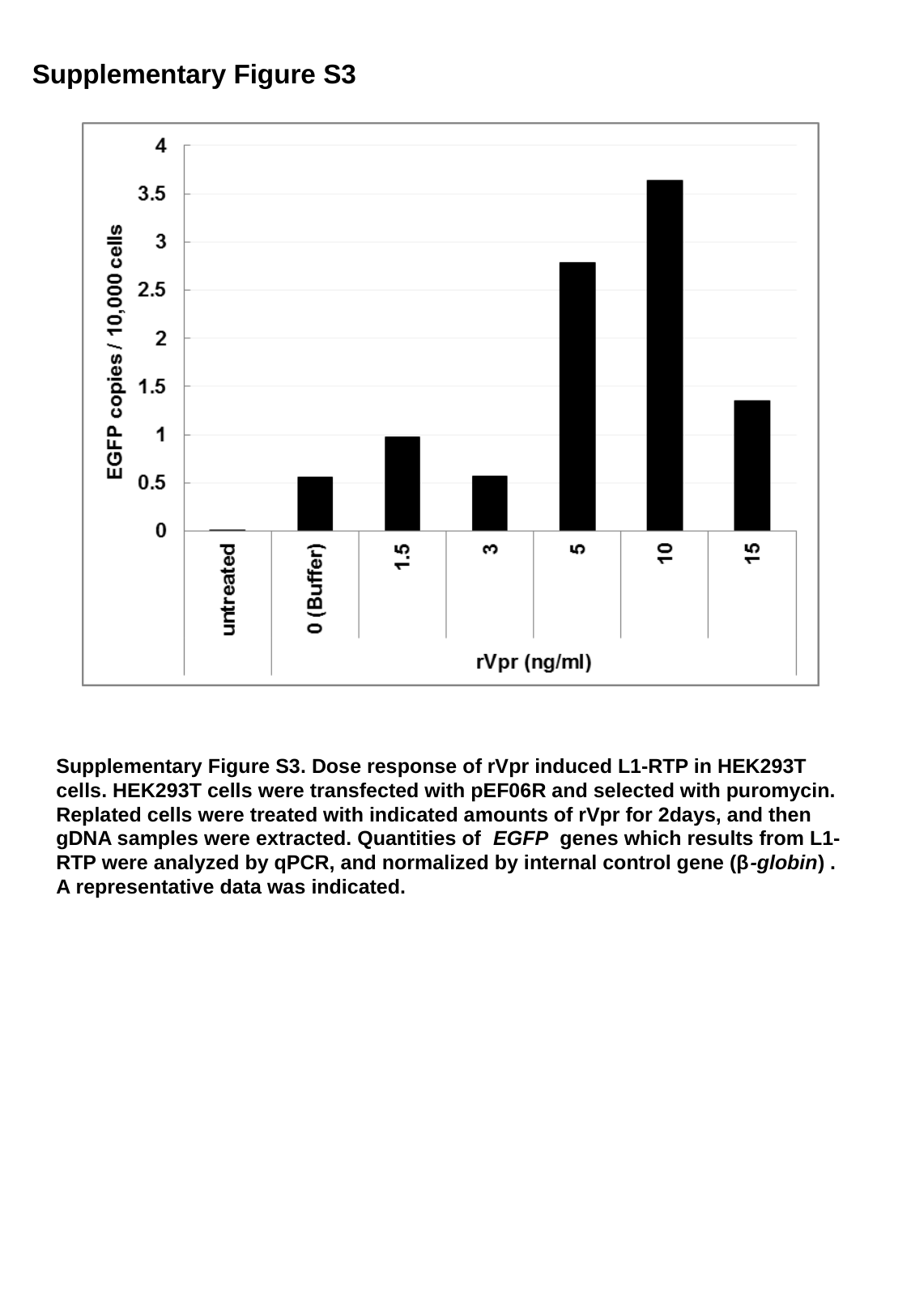

Supplementary Figure S3
Supplementary Figure S3. Dose response of rVpr induced L1-RTP in HEK293T cells. HEK293T cells were transfected with pEF06R and selected with puromycin. Replated cells were treated with indicated amounts of rVpr for 2days, and then gDNA samples were extracted. Quantities of EGFP genes which results from L1-RTP were analyzed by qPCR, and normalized by internal control gene (β-globin) . A representative data was indicated.
