# Supplementary material for: Viral protein R of human immunodeficiency virus type-1 induces retrotransposition of long interspersed element-1
Source: Retrovirology. 2013 Aug 5;10:83. doi: 10.1186/1742-4690-10-83 (PMC3751050; doi:10.1186/1742-4690-10-83)
Supplement: Additional file 4: Table S1 — Summary viral titres and L1-RTP activity. [file 1742-4690-10-83-S4.doc]

| Supplementary Table S1. Summary viral titres and L1-RTP activity | | | |
| --- | --- | --- | --- |
| Patient  number | CD4 count (cells/mL) | Viral load  (copies/mL) | L1-RTP  activity |
| 1 | 739 | 8700 | + |
| 2 | 607 | 230000 | + |
| 3 | 379 | 230000 | - |
| 4 | 591 | 23000 | - |
| 5 | 558 | 12000 | - |
| 6 | 387 | 5600 | - |
| 7 | 320 | 50 | + |
| 8 | 428 | 38000 | - |
| 9 | 750 | 820 | - |
| 10 | 598 | 620 | - |
| 11 | 795 | 410 | - |
| 12 | 510 | 400 | + |
| 13 | 384 | 1100 | - |
| 14 | 429 | 400 | + |
| 15 | 315 | 140 | + |

All patients were males.
