# Supplementary material for: Viral protein R of human immunodeficiency virus type-1 induces retrotransposition of long interspersed element-1
Source: Retrovirology. 2013 Aug 5;10:83. doi: 10.1186/1742-4690-10-83 (PMC3751050; doi:10.1186/1742-4690-10-83)
Supplement: Additional file 5: Figure S4 — Detection of Vpr in blood samples of HIV-1 positive patients. [file 1742-4690-10-83-S5.ppt]

## Slide 1
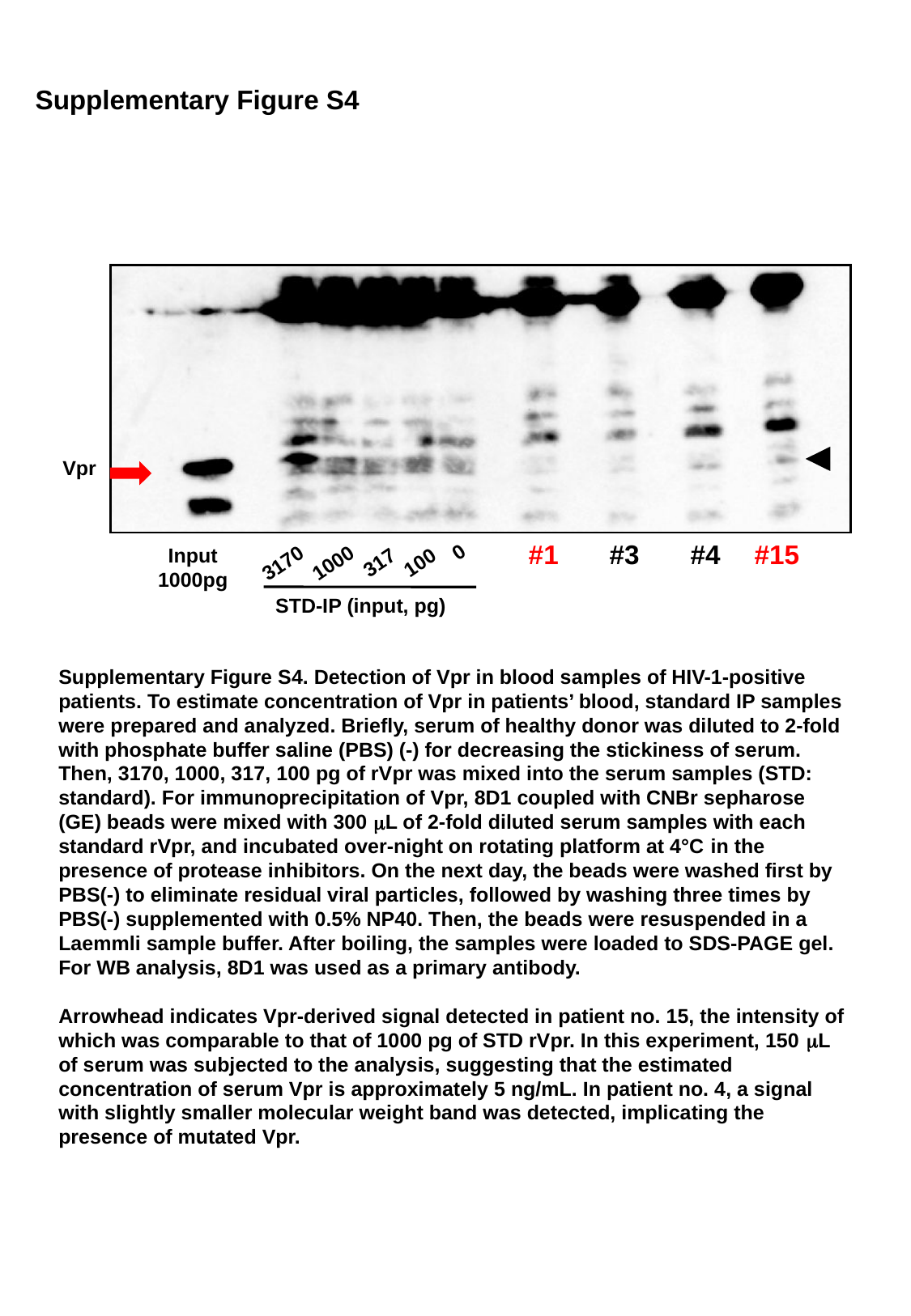

Supplementary Figure S4
Vpr
0
317
100
3170
1000
#1
#3
#4
#15
Input
1000pg
STD-IP (input, pg)
Supplementary Figure S4. Detection of Vpr in blood samples of HIV-1-positive patients. To estimate concentration of Vpr in patients’ blood, standard IP samples were prepared and analyzed. Briefly, serum of healthy donor was diluted to 2-fold with phosphate buffer saline (PBS) (-) for decreasing the stickiness of serum. Then, 3170, 1000, 317, 100 pg of rVpr was mixed into the serum samples (STD: standard). For immunoprecipitation of Vpr, 8D1 coupled with CNBr sepharose (GE) beads were mixed with 300 μL of 2-fold diluted serum samples with each standard rVpr, and incubated over-night on rotating platform at 4°C in the presence of protease inhibitors. On the next day, the beads were washed first by PBS(-) to eliminate residual viral particles, followed by washing three times by PBS(-) supplemented with 0.5% NP40. Then, the beads were resuspended in a Laemmli sample buffer. After boiling, the samples were loaded to SDS-PAGE gel. For WB analysis, 8D1 was used as a primary antibody.
Arrowhead indicates Vpr-derived signal detected in patient no. 15, the intensity of which was comparable to that of 1000 pg of STD rVpr. In this experiment, 150 μL of serum was subjected to the analysis, suggesting that the estimated concentration of serum Vpr is approximately 5 ng/mL. In patient no. 4, a signal with slightly smaller molecular weight band was detected, implicating the presence of mutated Vpr.
