# Supplementary material for: Viral protein R of human immunodeficiency virus type-1 induces retrotransposition of long interspersed element-1
Source: Retrovirology. 2013 Aug 5;10:83. doi: 10.1186/1742-4690-10-83 (PMC3751050; doi:10.1186/1742-4690-10-83)
Supplement: Additional file 6: Table S2 — Summary of the PCR-based assay in vivo. [file 1742-4690-10-83-S6.doc]

Supplementary Table S2. Summary of the PCR-based assay of organs positive for rVpr-induced L1-RTP in hL1-Tg mice.

| Mouse lines | No. mice | Organs | | | | |
| --- | --- | --- | --- | --- | --- | --- |
|  |  | Bone marrow | Thymus | Spleen | Lymph nodes | Kidney |
| #4 | 5 | 4 | 4 | 5 | 3 | 5 |
| #67 | 4 | 2 | 3 | 4 | 2 | 4 |
| Total | 9 | 6 | 7 | 9 | 5 | 9 |

rVpr was administered into hL1-EGFP mice three times of 100 or 200 ng.

Representative results of the PCR-based assay were shown in Figure 3.

Numbers of samples positive for L1-RTP with relative intensity (RI) more than

3-fold to control were counted.
