# Supplementary material for: Viral protein R of human immunodeficiency virus type-1 induces retrotransposition of long interspersed element-1
Source: Retrovirology. 2013 Aug 5;10:83. doi: 10.1186/1742-4690-10-83 (PMC3751050; doi:10.1186/1742-4690-10-83)
Supplement: Additional file 7: Figure S5 — Effects of d4T on rVpr-induced L1-RTP. [file 1742-4690-10-83-S7.ppt]

## Slide 1
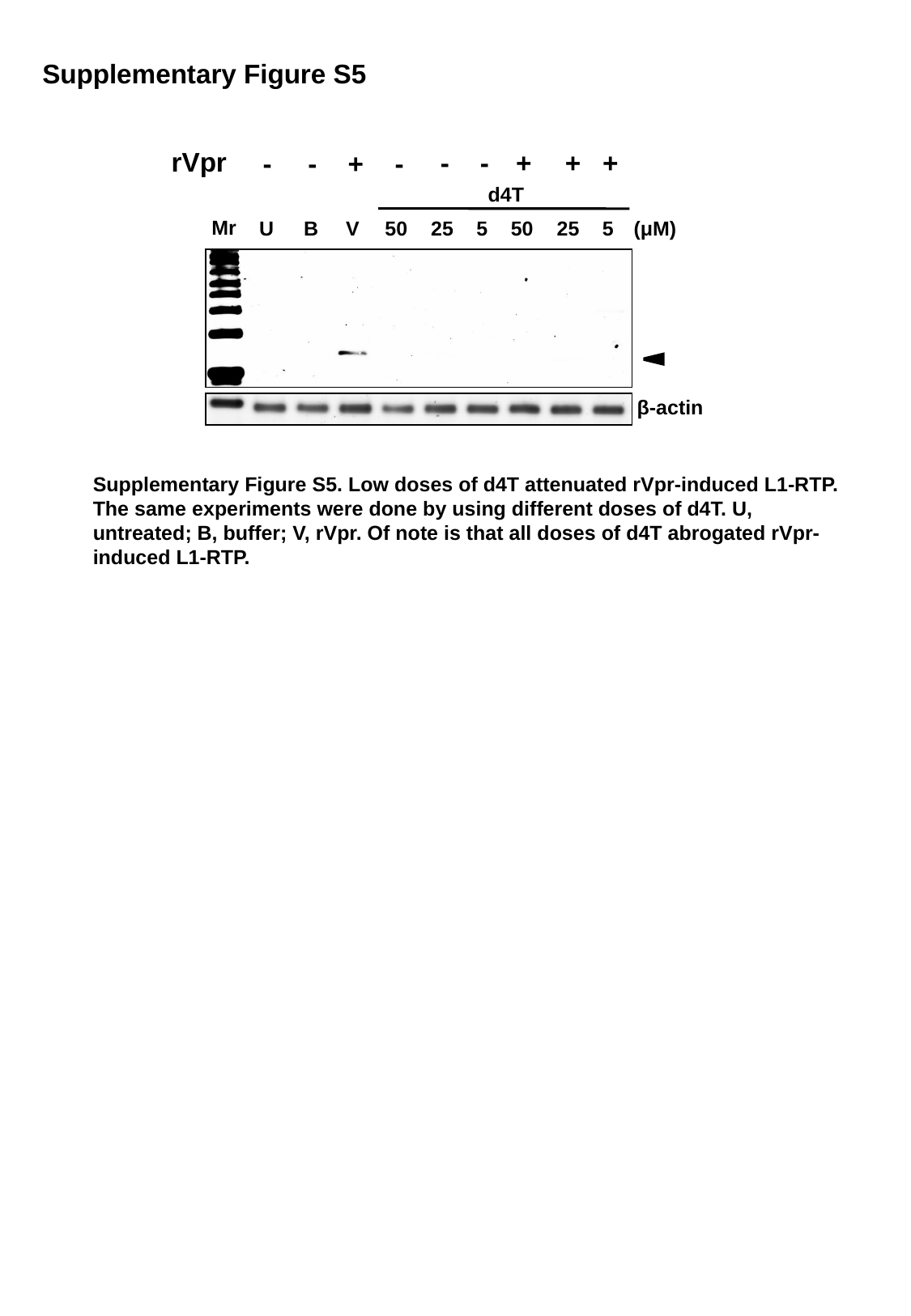

Supplementary Figure S5
rVpr
+
+
+
-
-
-
-
+
-
d4T
Mr
50
25
5
50
25
5
(μM)
U
B
V
β-actin
Supplementary Figure S5. Low doses of d4T attenuated rVpr-induced L1-RTP. The same experiments were done by using different doses of d4T. U, untreated; B, buffer; V, rVpr. Of note is that all doses of d4T abrogated rVpr-induced L1-RTP.
