# Supplementary material for: Viral protein R of human immunodeficiency virus type-1 induces retrotransposition of long interspersed element-1
Source: Retrovirology. 2013 Aug 5;10:83. doi: 10.1186/1742-4690-10-83 (PMC3751050; doi:10.1186/1742-4690-10-83)
Supplement: Additional file 9: Figure S7 — Inhibitory effects of MNF on rVpr-induced L1-RTP. [file 1742-4690-10-83-S9.ppt]

## Slide 1
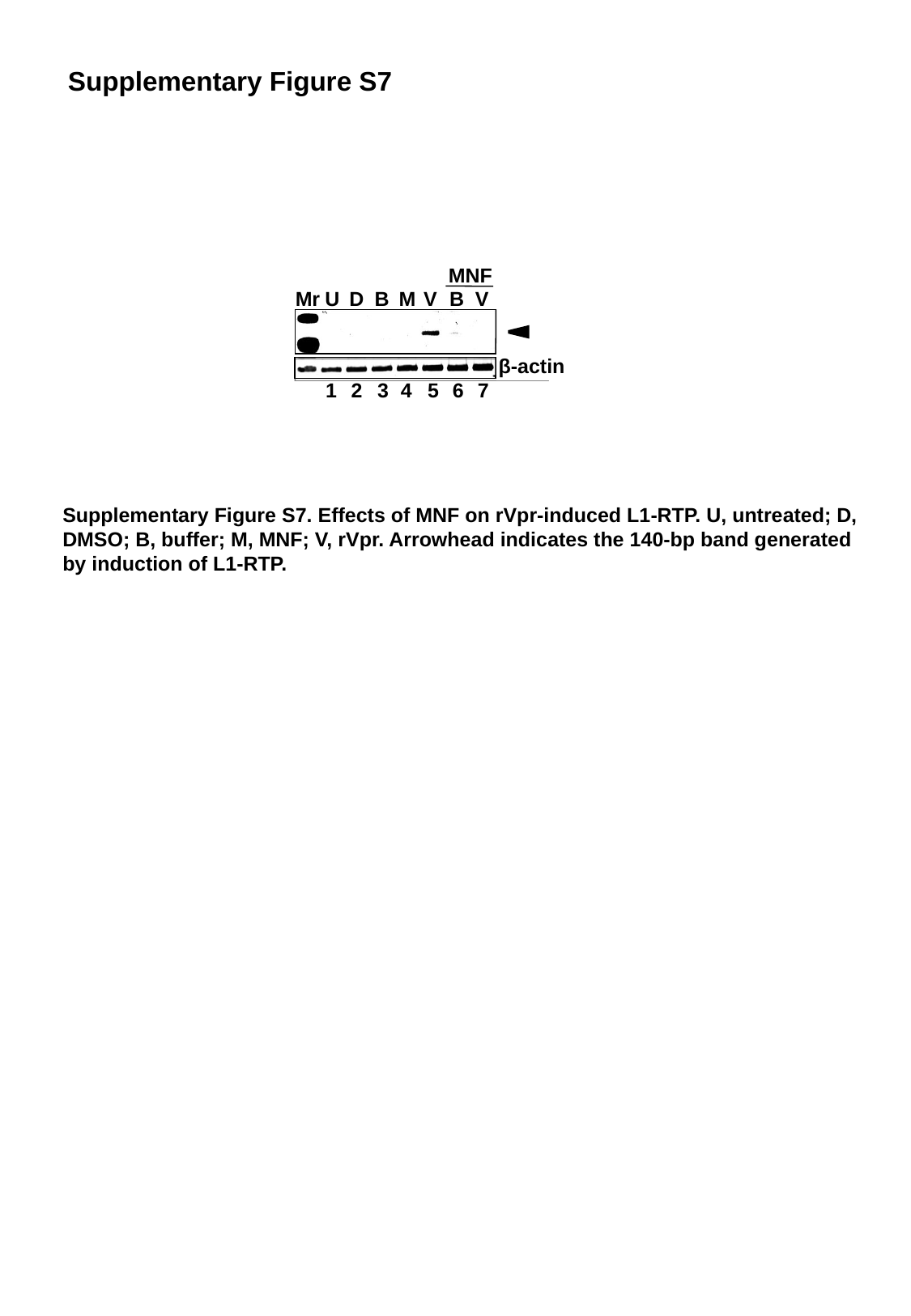

Supplementary Figure S7
MNF
Mr
U
D
B
B
V
M
V
β-actin
1
2
3
4
5
6
7
Supplementary Figure S7. Effects of MNF on rVpr-induced L1-RTP. U, untreated; D, DMSO; B, buffer; M, MNF; V, rVpr. Arrowhead indicates the 140-bp band generated by induction of L1-RTP.
