# Supplementary material for: Viral protein R of human immunodeficiency virus type-1 induces retrotransposition of long interspersed element-1
Source: Retrovirology. 2013 Aug 5;10:83. doi: 10.1186/1742-4690-10-83 (PMC3751050; doi:10.1186/1742-4690-10-83)
Supplement: Additional file 10: Figure S8 — Effects of siRNAs of AhR, ARNT1, CREB and c-Jun on expression of endogenous proteins. [file 1742-4690-10-83-S10.ppt]

## Slide 1
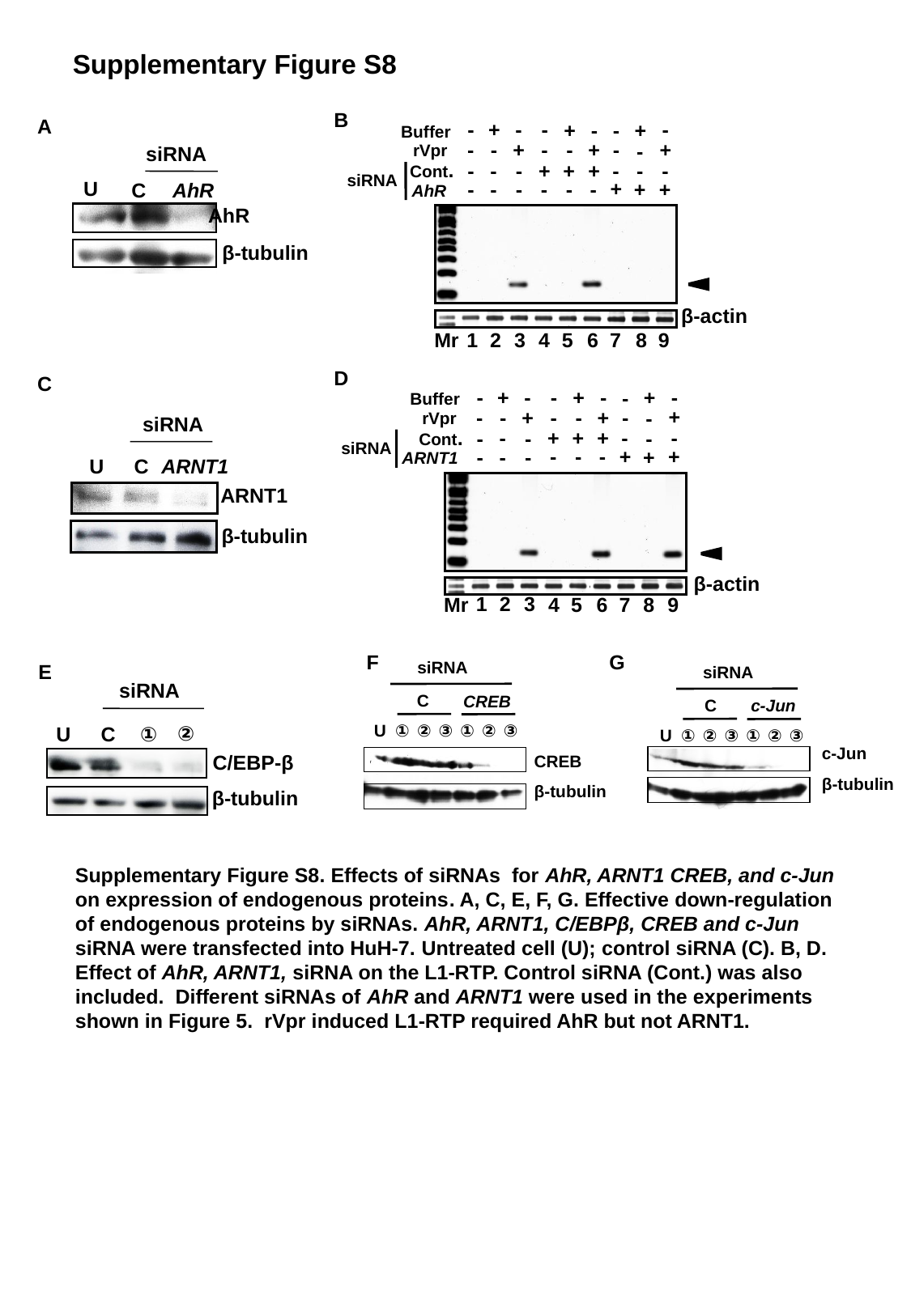

Supplementary Figure S8
B
A
+
-
-
-
-
-
+
+
-
Buffer
+
+
-
-
-
-
+
-
-
rVpr
siRNA
Cont.
-
+
+
+
-
-
-
-
-
siRNA
U
+
-
-
-
+
-
-
-
+
AhR
C
AhR
AhR
β-tubulin
β-actin
1
2
3
Mr
4
5
6
7
8
9
D
C
+
-
-
-
-
-
+
+
-
Buffer
+
+
-
-
-
-
+
-
-
rVpr
siRNA
Cont.
-
+
+
+
-
-
-
-
-
siRNA
+
-
-
-
+
-
-
-
+
ARNT1
C
ARNT1
U
ARNT1
β-tubulin
β-actin
1
2
3
Mr
4
5
6
7
8
9
F
G
siRNA
C
CREB
U
①
②
③
①
②
③
CREB
β-tubulin
E
siRNA
C
c-Jun
U
①
②
③
①
②
③
c-Jun
β-tubulin
siRNA
②
U
C
①
C/EBP-β
β-tubulin
Supplementary Figure S8. Effects of siRNAs for AhR, ARNT1 CREB, and c-Jun on expression of endogenous proteins. A, C, E, F, G. Effective down-regulation of endogenous proteins by siRNAs. AhR, ARNT1, C/EBPβ, CREB and c-Jun siRNA were transfected into HuH-7. Untreated cell (U); control siRNA (C). B, D. Effect of AhR, ARNT1, siRNA on the L1-RTP. Control siRNA (Cont.) was also included. Different siRNAs of AhR and ARNT1 were used in the experiments shown in Figure 5. rVpr induced L1-RTP required AhR but not ARNT1.
