# Supplementary material for: Viral protein R of human immunodeficiency virus type-1 induces retrotransposition of long interspersed element-1
Source: Retrovirology. 2013 Aug 5;10:83. doi: 10.1186/1742-4690-10-83 (PMC3751050; doi:10.1186/1742-4690-10-83)
Supplement: Additional file 11: Figure S9 — CYP1A1 expression under down-regulation of AhR or ARNT1. [file 1742-4690-10-83-S11.ppt]

## Slide 1
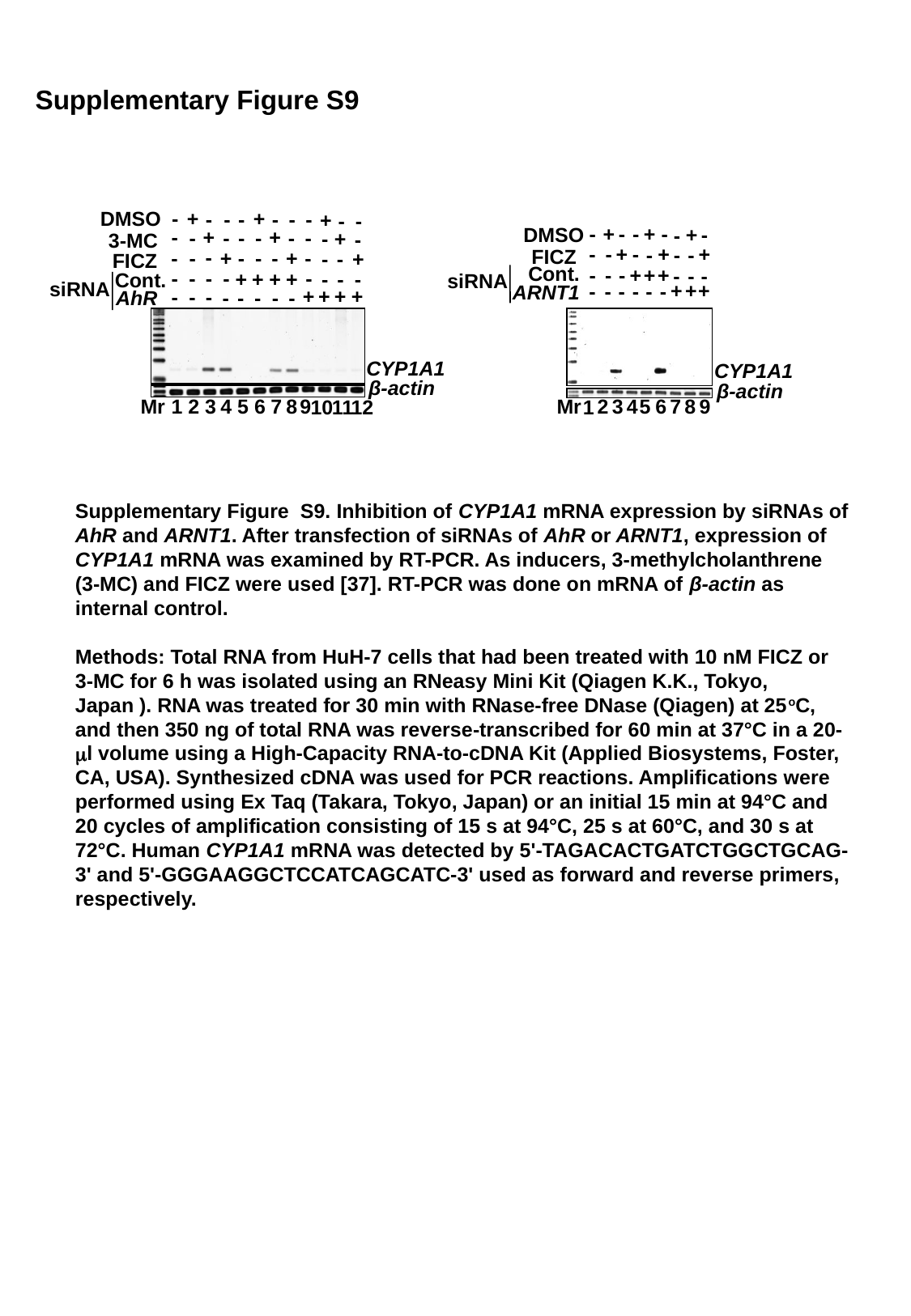

Supplementary Figure S9
+
DMSO
+
-
-
-
-
-
-
-
+
-
-
+
-
-
-
+
-
+
DMSO
-
-
+
-
+
-
-
-
-
-
-
+
-
-
3-MC
+
-
-
+
-
+
-
-
-
FICZ
-
-
-
+
-
-
-
+
-
-
-
+
FICZ
Cont.
-
-
-
+
+
+
-
-
-
-
-
-
-
-
+
+
-
-
+
+
-
Cont.
siRNA
siRNA
+
+
+
-
-
-
-
-
-
ARNT1
+
+
+
+
-
-
-
AhR
-
-
-
-
-
CYP1A1
CYP1A1
β-actin
β-actin
1
2
3
4
5
4
5
6
7
8
9
Mr
Mr
6
7
8
9
2
3
10
1
11
12
Supplementary Figure S9. Inhibition of CYP1A1 mRNA expression by siRNAs of AhR and ARNT1. After transfection of siRNAs of AhR or ARNT1, expression of CYP1A1 mRNA was examined by RT-PCR. As inducers, 3-methylcholanthrene (3-MC) and FICZ were used [37]. RT-PCR was done on mRNA of β-actin as internal control.
Methods: Total RNA from HuH-7 cells that had been treated with 10 nM FICZ or 3-MC for 6 h was isolated using an RNeasy Mini Kit (Qiagen K.K., Tokyo, Japan ). RNA was treated for 30 min with RNase-free DNase (Qiagen) at 25oC, and then 350 ng of total RNA was reverse-transcribed for 60 min at 37°C in a 20-μl volume using a High-Capacity RNA-to-cDNA Kit (Applied Biosystems, Foster, CA, USA). Synthesized cDNA was used for PCR reactions. Amplifications were performed using Ex Taq (Takara, Tokyo, Japan) or an initial 15 min at 94°C and 20 cycles of amplification consisting of 15 s at 94°C, 25 s at 60°C, and 30 s at 72°C. Human CYP1A1 mRNA was detected by 5'-TAGACACTGATCTGGCTGCAG-3' and 5'-GGGAAGGCTCCATCAGCATC-3' used as forward and reverse primers, respectively.
