# Supplementary material for: Viral protein R of human immunodeficiency virus type-1 induces retrotransposition of long interspersed element-1
Source: Retrovirology. 2013 Aug 5;10:83. doi: 10.1186/1742-4690-10-83 (PMC3751050; doi:10.1186/1742-4690-10-83)
Supplement: Additional file 12: Figure S10 — Effects of MAPK inhibitors on rVpr-induced L1-RTP. [file 1742-4690-10-83-S12.ppt]

## Slide 1
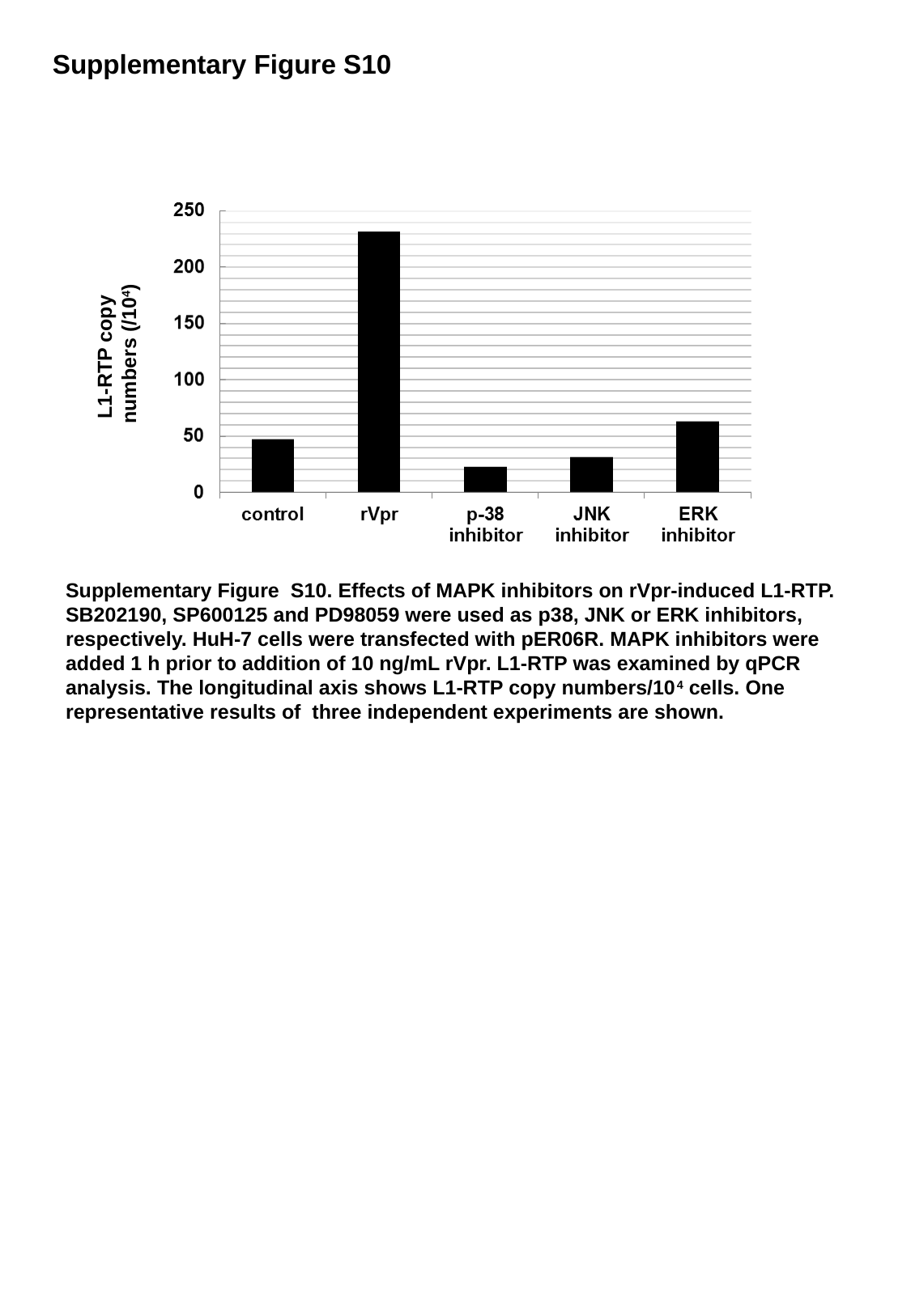

Supplementary Figure S10
L1-RTP copy
numbers (/104)
Supplementary Figure S10. Effects of MAPK inhibitors on rVpr-induced L1-RTP. SB202190, SP600125 and PD98059 were used as p38, JNK or ERK inhibitors, respectively. HuH-7 cells were transfected with pER06R. MAPK inhibitors were added 1 h prior to addition of 10 ng/mL rVpr. L1-RTP was examined by qPCR analysis. The longitudinal axis shows L1-RTP copy numbers/104 cells. One representative results of three independent experiments are shown.
