# Supplementary material for: Viral protein R of human immunodeficiency virus type-1 induces retrotransposition of long interspersed element-1
Source: Retrovirology. 2013 Aug 5;10:83. doi: 10.1186/1742-4690-10-83 (PMC3751050; doi:10.1186/1742-4690-10-83)
Supplement: Additional file 13: Figure S11 — Effects of AhR siRNA on chromatin recruitment of ORF1. [file 1742-4690-10-83-S13.ppt]

## Slide 1
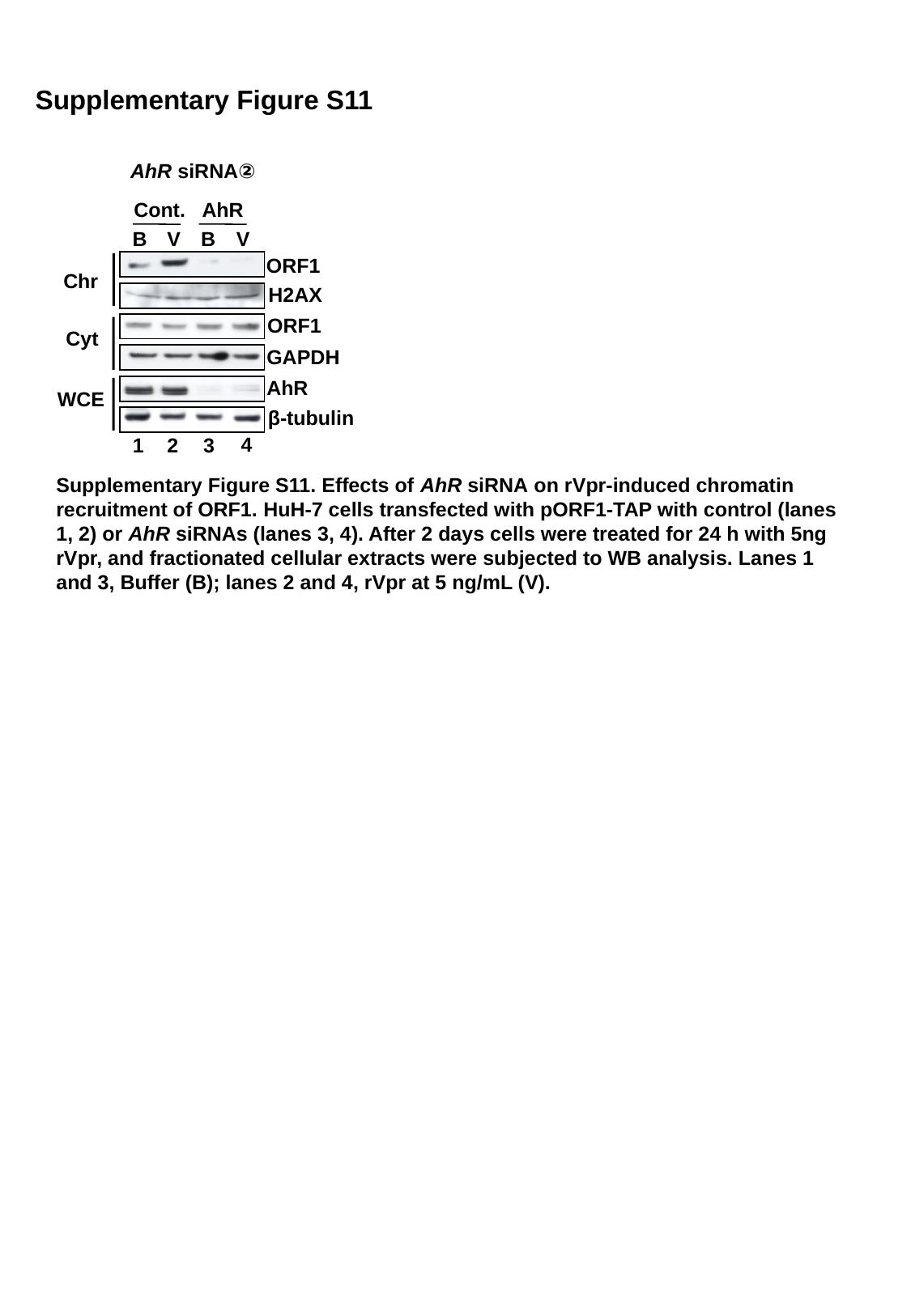

Supplementary Figure S11
AhR siRNA②
Cont.
AhR
B
V
B
V
ORF1
Chr
H2AX
ORF1
Cyt
GAPDH
AhR
WCE
β-tubulin
4
1
2
3
Supplementary Figure S11. Effects of AhR siRNA on rVpr-induced chromatin recruitment of ORF1. HuH-7 cells transfected with pORF1-TAP with control (lanes 1, 2) or AhR siRNAs (lanes 3, 4). After 2 days cells were treated for 24 h with 5ng rVpr, and fractionated cellular extracts were subjected to WB analysis. Lanes 1 and 3, Buffer (B); lanes 2 and 4, rVpr at 5 ng/mL (V).
