# Supplementary material for: Viral protein R of human immunodeficiency virus type-1 induces retrotransposition of long interspersed element-1
Source: Retrovirology. 2013 Aug 5;10:83. doi: 10.1186/1742-4690-10-83 (PMC3751050; doi:10.1186/1742-4690-10-83)
Supplement: Additional file 14: Figure S12 — Constitutive association of ORF1 and AhR under the conditions competent for the induction of L1-RTP. [file 1742-4690-10-83-S14.ppt]

## Slide 1
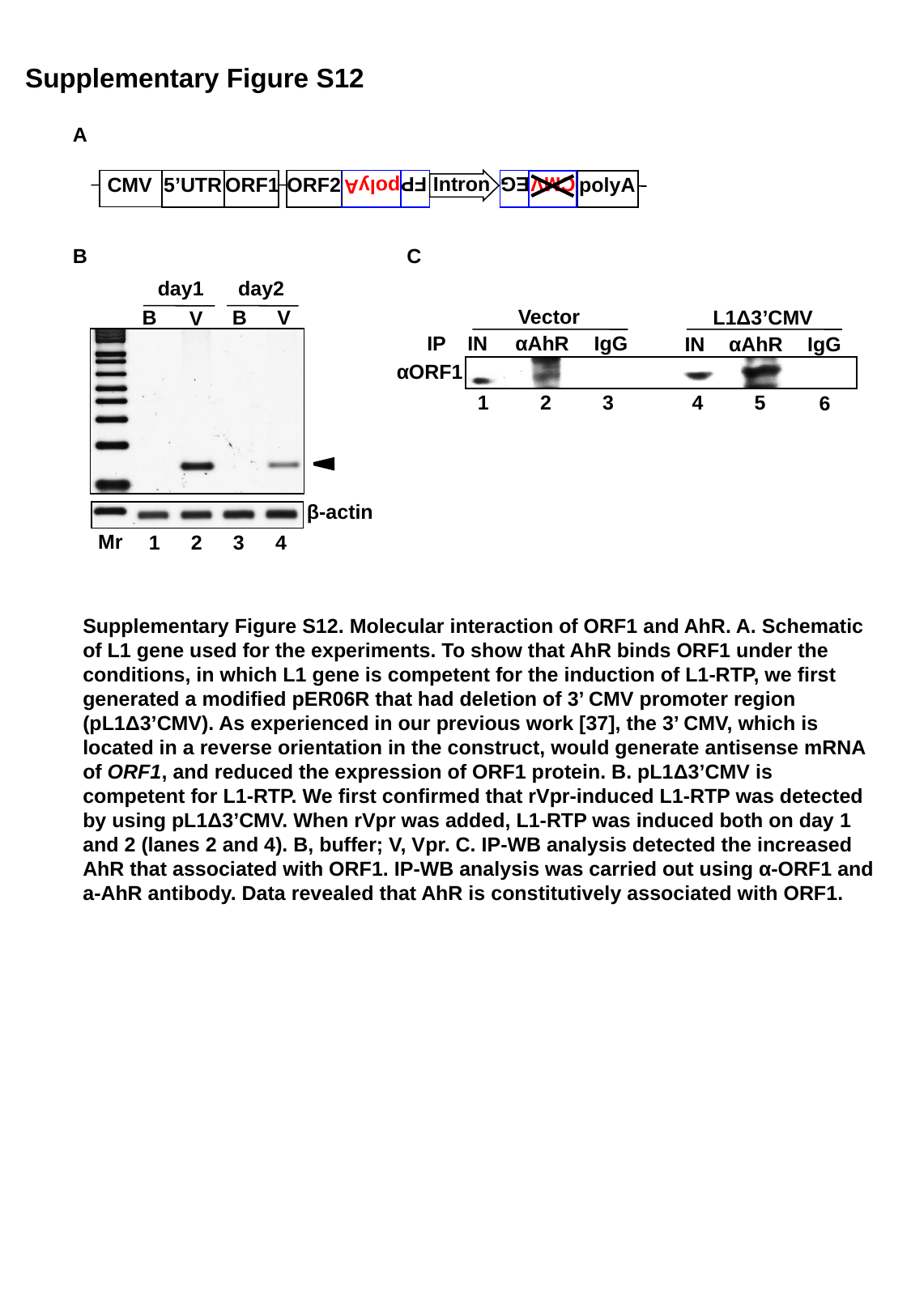

Supplementary Figure S12
A
Intron
polyA
ORF1
ORF2
CMV
5’UTR
EG FP
CMV
polyA
B
C
day1
day2
Vector
B
L1Δ3’CMV
B
V
V
IP
IN
αAhR
IgG
IN
αAhR
IgG
αORF1
1
2
3
4
5
6
β-actin
Mr
1
2
3
4
Supplementary Figure S12. Molecular interaction of ORF1 and AhR. A. Schematic of L1 gene used for the experiments. To show that AhR binds ORF1 under the conditions, in which L1 gene is competent for the induction of L1-RTP, we first generated a modified pER06R that had deletion of 3’ CMV promoter region (pL1Δ3’CMV). As experienced in our previous work [37], the 3’ CMV, which is located in a reverse orientation in the construct, would generate antisense mRNA of ORF1, and reduced the expression of ORF1 protein. B. pL1Δ3’CMV is competent for L1-RTP. We first confirmed that rVpr-induced L1-RTP was detected by using pL1Δ3’CMV. When rVpr was added, L1-RTP was induced both on day 1 and 2 (lanes 2 and 4). B, buffer; V, Vpr. C. IP-WB analysis detected the increased AhR that associated with ORF1. IP-WB analysis was carried out using α-ORF1 and a-AhR antibody. Data revealed that AhR is constitutively associated with ORF1.
