# Supplementary material for: Viral protein R of human immunodeficiency virus type-1 induces retrotransposition of long interspersed element-1
Source: Retrovirology. 2013 Aug 5;10:83. doi: 10.1186/1742-4690-10-83 (PMC3751050; doi:10.1186/1742-4690-10-83)
Supplement: Additional file 16: Figure S14 — L1-RTP by Vpr required a carboxy-terminal region. [file 1742-4690-10-83-S16.ppt]

## Slide 1
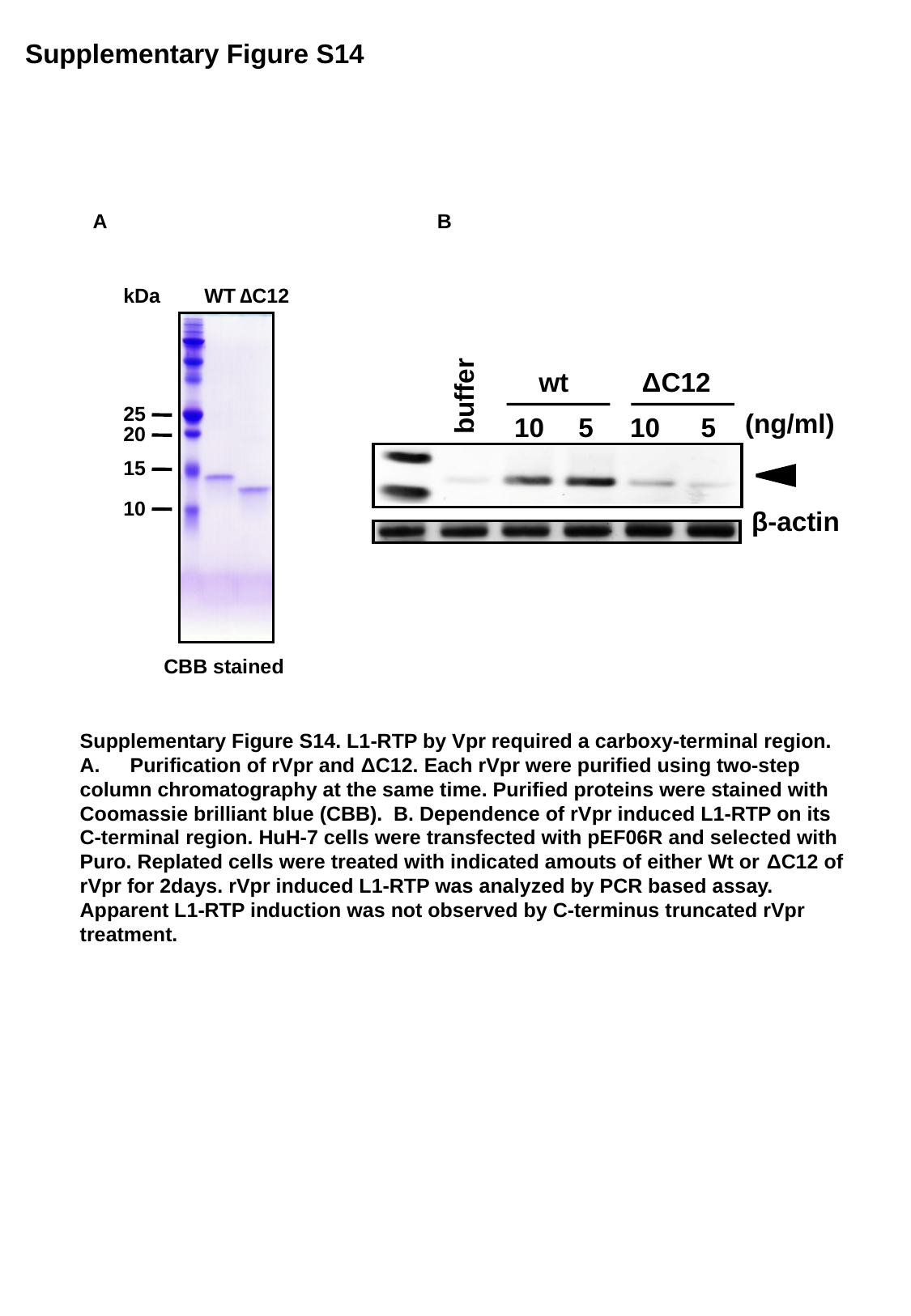

Supplementary Figure S14
A
B
kDa
WT
∆C12
buffer
wt
ΔC12
(ng/ml)
10
5
10
5
β-actin
25
20
15
10
CBB stained
Supplementary Figure S14. L1-RTP by Vpr required a carboxy-terminal region.
A.　Purification of rVpr and ΔC12. Each rVpr were purified using two-step column chromatography at the same time. Purified proteins were stained with Coomassie brilliant blue (CBB). B. Dependence of rVpr induced L1-RTP on its C-terminal region. HuH-7 cells were transfected with pEF06R and selected with Puro. Replated cells were treated with indicated amouts of either Wt or ΔC12 of rVpr for 2days. rVpr induced L1-RTP was analyzed by PCR based assay. Apparent L1-RTP induction was not observed by C-terminus truncated rVpr treatment.
