# Supplementary material for: Viral protein R of human immunodeficiency virus type-1 induces retrotransposition of long interspersed element-1
Source: Retrovirology. 2013 Aug 5;10:83. doi: 10.1186/1742-4690-10-83 (PMC3751050; doi:10.1186/1742-4690-10-83)
Supplement: Additional file 17: Figure S15 — Effects of benzonase on the interaction of AhR and ORF1 or Vpr. [file 1742-4690-10-83-S17.ppt]

## Slide 1
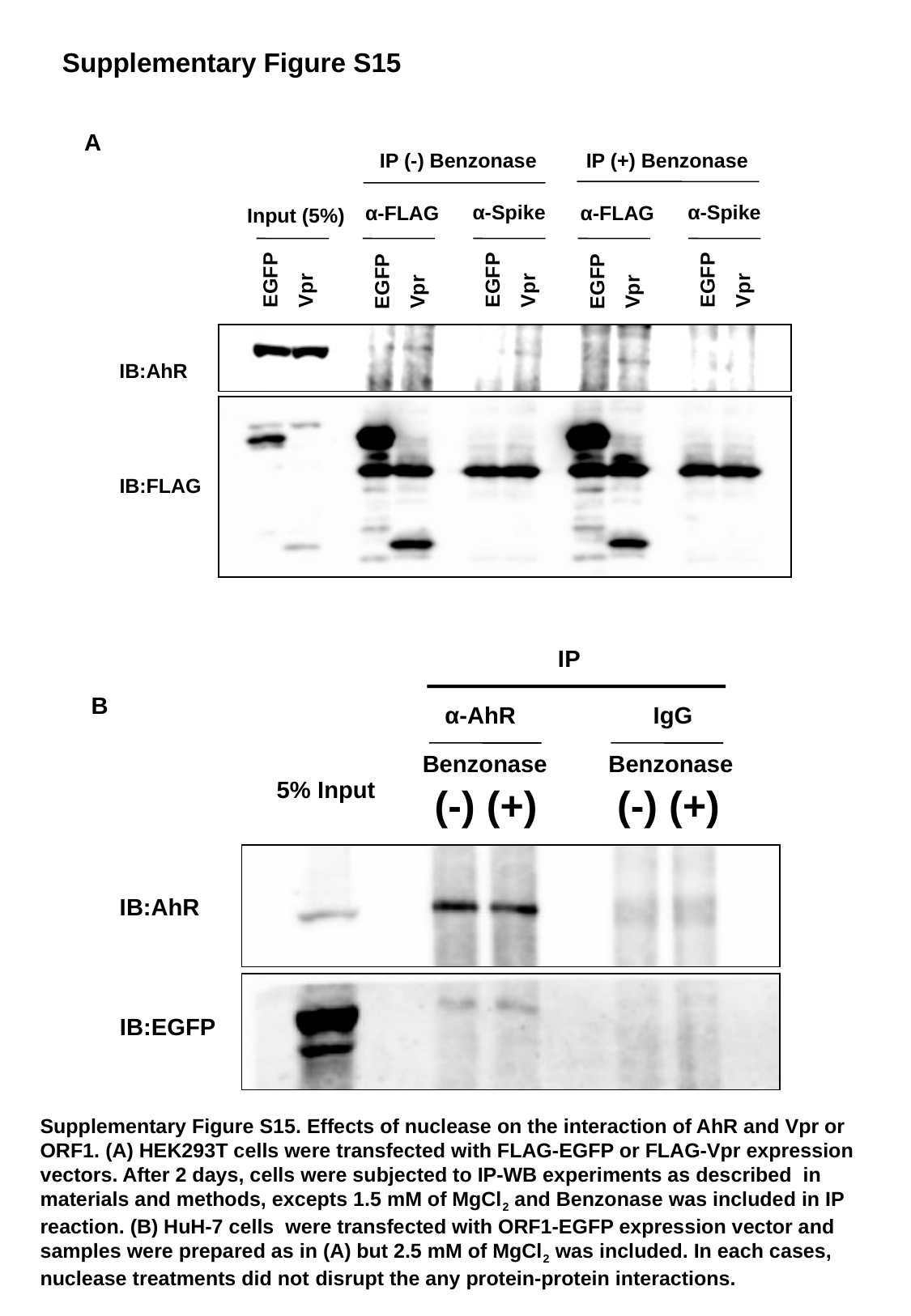

Supplementary Figure S15
A
IP (-) Benzonase
IP (+) Benzonase
α-Spike
α-Spike
α-FLAG
α-FLAG
Input (5%)
EGFP
EGFP
EGFP
EGFP
EGFP
Vpr
Vpr
Vpr
Vpr
Vpr
IB:AhR
IB:FLAG
IP
B
α-AhR
IgG
Benzonase
Benzonase
5% Input
(-) (+)
(-) (+)
IB:AhR
IB:EGFP
Supplementary Figure S15. Effects of nuclease on the interaction of AhR and Vpr or ORF1. (A) HEK293T cells were transfected with FLAG-EGFP or FLAG-Vpr expression vectors. After 2 days, cells were subjected to IP-WB experiments as described in materials and methods, excepts 1.5 mM of MgCl2 and Benzonase was included in IP reaction. (B) HuH-7 cells were transfected with ORF1-EGFP expression vector and samples were prepared as in (A) but 2.5 mM of MgCl2 was included. In each cases, nuclease treatments did not disrupt the any protein-protein interactions.
