# Supplementary material for: Viral protein R of human immunodeficiency virus type-1 induces retrotransposition of long interspersed element-1
Source: Retrovirology. 2013 Aug 5;10:83. doi: 10.1186/1742-4690-10-83 (PMC3751050; doi:10.1186/1742-4690-10-83)
Supplement: Additional file 18: Table S3 — Nucleotide sequence of siRNA used in the current study. [file 1742-4690-10-83-S18.doc]

Supplementary Table S3. Nucleotide sequence of siRNA used in the current study.

| Genes |  | Nucleotide sequences (5'-3') |
| --- | --- | --- |
| *AhR* -1 | Se | GCAUGAUAGUUUUCCGGCUTT |
|  | AS | AGCCGGAAAACUAUCAUGCCA |
| -2 | Se | GGCUCUUUCAAGAUAGUAATT |
|  | AS | UUACUAUCUUGAAAGAGCCCT |
| *ARNT1* -1 | Se | GGCGUAUCCUGGAUCUAAATT |
|  | AS | UUUAGAUCCAGGAUACGCCCT |
| -2 | Se | CAGUUUCUGUGAAUAGGCUTT |
|  | AS | AGCCUAUUCACAGAAACUGGG |
| *C/EBP-β* -1 | Se | GCCCGUCGGUAAUUUUAAUTT |
|  | AS | AUUAAAAUUACCGACGGGCTC |
| -2 | Se | CCAACCGCACAUGCAGAUGTT |
|  | AS | CAUCUGCAUGUGCGGUUGGTT |
| *CREB* -1 | Se | GCUGGCUAACAAUGGUACCTT |
|  | AS | GGUACCAUUGUUAGCCAGCTG |
| -2 | Se | CCAAUCCCUUGAGUUAUAUTT |
|  | AS | AUAUAACUCAAGGGAUUGGTT |
| *c-Jun* -1 | Se | GGCACAGCUUAAACAGAAATT |
|  | AS | UUUCUGUUUAAGCUGUGCCAC |
| -2 | Se | GGAUCAAGGCGGAGAGGAATT |
|  | AS | UUCCUCUCCGCCUUGAUCCGC |

Nucleotide sequences of both sense (Se) and antisense (AS) strands were shown.
